# Scanning and three-dimensional-printing using computed tomography of the “Golden Boy” mummy

**DOI:** 10.3389/fmed.2022.1028377

**Published:** 2023-01-24

**Authors:** Sahar N. Saleem, Sabah Abd el-Razek Seddik, Mahmoud el-Halwagy

**Affiliations:** ^1^Department of Radiology, Kasr Al Ainy Faculty of Medicine, Cairo University, Cairo, Egypt; ^2^The Egyptian Museum, Cairo, Egypt

**Keywords:** Egypt, mummy, computed tomography, amulets, child, 3D printing

## Abstract

Ancient Egyptian mummies represent an opportunity to learn more about the health, beliefs, and skills of humans in antiquity. A fully wrapped mummy, from a Late Ptolemaic cemetery (c.332-30 BC) in Edfu, Egypt, has been stored, unexamined, at the Cairo Egyptian Museum since 1916. We hypothesized that scanning and 3D-printing the mummy using Computed Tomography (CT) could help in documenting and promoting its public display. CT enabled non-invasive digital unwrapping and revealed a well-preserved mummy. Biological sex could be determined from the presence of male genitalia; epiphyseal fusion and tooth eruption indicated an approximate age at death of 14–15 years. The deceased had healthy teeth and bones without evidence of poor nutrition or disease. CT detected a high-quality mummification process that included brain removal through an iatrogenic defect of the cribriform plate and viscera removal *via* a left lower-abdominal incision. The heart remained in the chest as a spiritual symbol. Resin was poured into the emptied cranial and torso cavities, and linen packs were placed inside the torso. The Mummy’s external ornamentation includes a gilded head mask, a pectoral cartonnage, and a pair of sandals. CT identified 49 amulets inside the mummy and between the wrappings, arranged in three columns. The amulets have 21 different shapes, including Udjat, scarabs, Ajet, Djed-pillar, Tyt, Placenta, Double-Plume, and Right-angle. CT densities indicated that 30 (61%) amulets were metal (likely gold), and the other amulets were made of faience, stones, or fired clay. The embalmers placed amulets to protect and provide vitality for the body for the afterlife. A gold tongue amulet was placed inside the mouth to ensure the deceased could speak in the afterlife. A Two-finger amulet was placed beside the penis to protect the embalming incision. 3D-printing enabled the tactile and visual study of a heart scarab found inside the thoracic cavity. Findings from this study suggest that ancient Egyptians valued their children and provided them with ritual treatment. State-of-the-art techniques such as CT and 3D printing provided valuable insights and supported the museum display of the mummy, nicknamed “The Golden Boy.”

## 1. Introduction

Egypt witnessed extensive excavations in the 19th and early 20th centuries that resulted in the unearthing of thousands of ancient, preserved bodies, many were still wrapped within their original coffins (1). Since its opening in 1835, the Egyptian Museum in Cairo has served as the repository of the excavation finds. The museum basement is crowded with many of these mummies that have been locked in for decades without being studied or ever displayed (2).

Ancient Egyptian mummies represent an opportunity to learn more about ancient human health, beliefs, and skills (1). In the past, mummies were unwrapped and subjected to invasive dissection for research and entertainment (1, 3). Finding an ideal compromise between investigating a mummy and not destroying it encouraged the use of less invasive methods. Radiology is a non-invasive method that has been used in mummy research since shortly after the discovery of x-rays in 1895 (4, 5). X-rays can discriminate different densities related to the mummy, such as bone, dense embalming materials, jewelry, and amulets (4). However, the overlapping of the three-dimensional (3D) data of the object on the two-dimensional (2D) x-ray film leads to loss of data and less satisfactory results. Computed Tomography (CT) represents a significant advance in radiology. Instead of using a single image, hundreds of projections of thin sections (slices) of the body can be combined to create a complete three-dimensional model of the body (5).

Ancient Egyptian mummies are cultural heritage that needs documentation, preservation, and display using updated technological applications (5–9). CT technology helped in non-invasive investigation and unwrapping ancient Egyptian mummies (6). Studies using CT provided valuable information about the mummy’s health and mummification style (6, 8). 3D-printing using different materials has been increasingly used in studying archeological artifacts. CT data can provide the three-dimensional model of an object needed for 3D-printing (7).

In this study, we hypothesized that CT scanning and 3D printing using CT data could help in studying and documenting a fully wrapped mummy stored in the basement of the Cairo Egyptian Museum. We hypothesized that this study would provide information about the mummy that could help in preservation and promote display of the mummy in the museum’s showroom.

## 2. Materials and methods

A wrapped mummy was housed at the time of this study at the storage area in the basement of The Egyptian Museum in Cairo in Location SS 17B under the code: CG 31153/TR 21/11/16/16, SR 11413. According to the registration book, the museum acquired this object on the 21st of November 1916. The object was found in Edfu (Apollinopolis Magna; Djeba; Msen)-Aswan governate, at site Nag el–Hassaya, in East Late Ptolemaic (LP) cemetery (circa 305-30 BC). The wrapped mummy is placed inside an outer and inner coffin. The outer coffin is a rectangular non-decorated wooden coffin with a lid. The outer coffin is barrel arch shaped and has Greek letters incised in black (Supplementary Figure 1). The inner wooden coffin is anthropoid in shape with gilded face and colored drawings in the style of late Ptolemaic era (Supplementary Figure 2).

### 2.1. CT scanning of the wrapped mummy

We moved the mummy from the storeroom to the garden of the museum for scanning using the Computed Tomography (CT) scanning machine (Somatom Emotion 6; Siemens Medical Solutions, Malvern, PA, USA) mounted on a truck there (Supplementary Figure 3). We used the following CT parameters: kV = 130; effective mAs: 23–63; and pitch: (0⋅83–1⋅8). We used FOV: 350–500; slice thickness: 0⋅6–1⋅25 mm; and reconstruction thickness: 0⋅4–0⋅8 mm. CT images were reconstructed with various convolution kernels. Axial images were generated, and we used OsiriX, reconstruction software (Pixmeo SARL, Bernex, Switzerland) to produce two- and three-dimensional CT images in MIP (Maximum Intensity Projection), MPR (Multi-Planar Reconstruction), SSD (Surface Shaded Display), and VRT (Volume Rendering Technique). We virtually unwrapped the mummy using CT scan using a protocol published previously (6). The mummy’s CT images were analyzed to determine the preservation status, sex, age at death, pathological changes and mummification using previously published protocols (5, 10–13). The age at death was estimated based on dental development, long bones development and epiphyseal fusion (10).

We obtained metric measurement (in mm) and measured the CT density of the mummy tissues in Hounsfield units (HU) using region of interest (ROI) tool. We correlated the findings with the results of the tests as well as the available archeological data and literature.

### 2.2. Foreign objects and amulets related to the wrapped mummy

We recorded the following for each foreign object/amulet within the mummy and its wrappings: its location within the mummy, metric measurements, shape, and CT densities value in Hounsfield units (HU) (14). We suggested the material of the object/amulet by its CT density (HU). The range of the CT densities in the scanner we used was limited to (-1,024 to +3,071 HU). Objects measuring the maximum CT density in this scale (3,071 HU) were considered (>3,071 HU). Metals were identified when an object with a high attenuation value >3,000 HU; stones (2,500–2,900 HU); Quartz/Faience (1,693–2,317 HU); and fired clay (1,116 HU SD 54.7) (14–16).

We identified amulets according to their shape (iconography) (Supplementary Table 1) in correlation with archeological references and with photographs of similar objects placed at Cairo Egyptian Museum or on the website of other world museums (1, 3). Resin or resin-like substance are identified according to CT findings as previously described: featureless homogeneous moderately dense (70–100 HU) structure may appear with a fluid level (5).

### 2.3. Three-dimensional (3D) printing of an amulet inside the wrapped mummy

The result of the 3D CT scanning of a selected object (the largest amulet in the mummy) was translated to a printable format. We removed the reconstructed signal that did not relate to the object. We changed the CT image format (DICOM, Digital Imaging and Communications in Medicine) to a volume file STL (Standard Tessellation Language/STereoLithography) format. Using a software (3DSlicer)^1^, we manipulated the image using built in segmentation tools. We segmented the required part (amulet) to get the surface hull. We used a commercial 3D printer (Dremel 3D Ideal Builder), a white ABS (acrylonitrile butadiene styrene) plastic material. The 3D model derived from the CT data was printed with a layer thickness of 0.1 mm using the fused deposit printing procedure.

### 2.4. Preparation for museum exhibition

We generated 2D- and 3D-CT images of the mummy to be used in a short documentary movie to be displayed beside the mummy in a museum showroom. 3D printed object to be used for tactile and visual experience by the visitors.

## 3. Results

### 3.1. Physical inspection of the mummy

**FIGURE 1:**
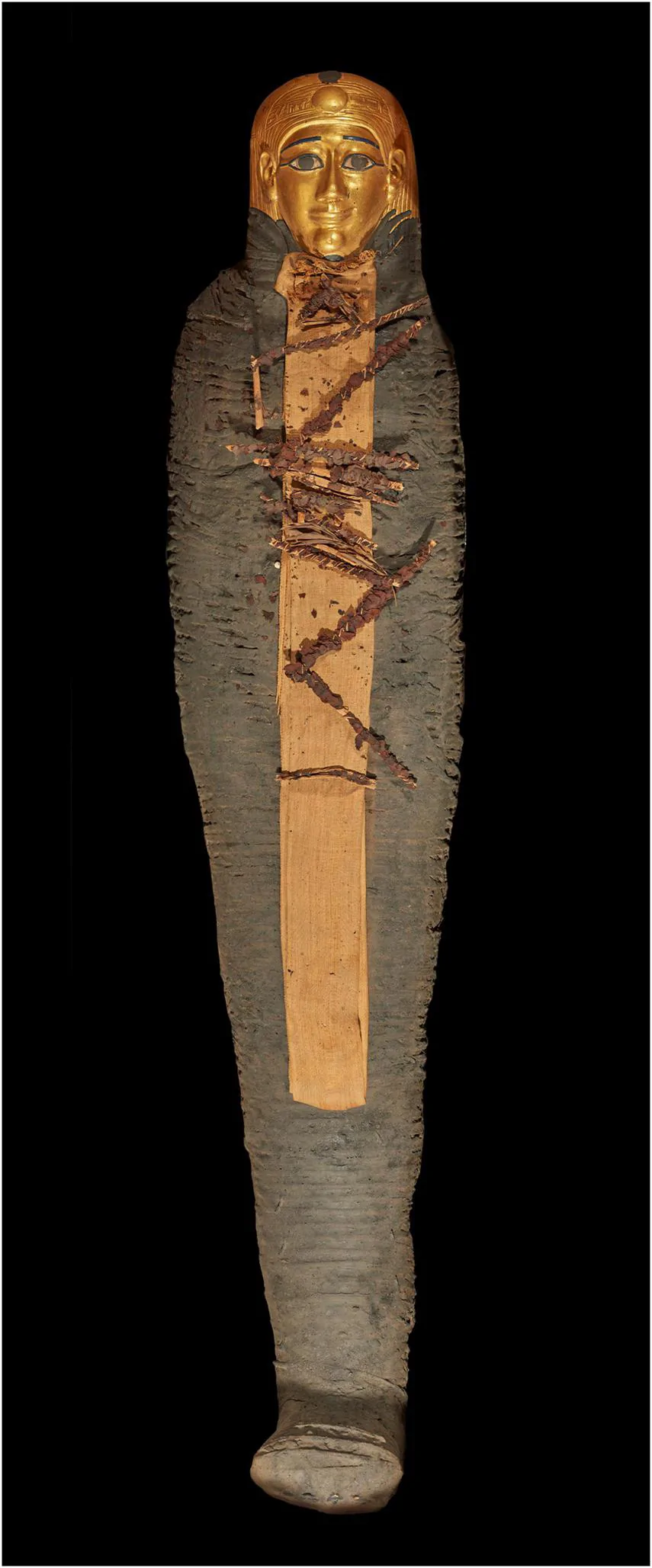
Front view photo of the mummy coded TR 21/11/16/16 at the Cairo Egyptian Museum. The mummy wears a gilded head mask, and the body is fully covered by brown-colored wrapping. Dried ferns are placed on a light beige rectangular linen on the front surface of the body.

The mummy wears a golden head mask with idealized facial features appropriate to the custom of the time. The eye lines and eyebrows are inlaid with stones, and the eye pupil is made of black obsidian. The mummy is fully wrapped from neck to feet. The outer linen of the wrapping is dark brown. A rectangular light brown linen sheet with a width of 88 mm placed on the middle of the anterior surface of the mummy extends from the neck to the level of the legs of the mummy. The sheet measures 88 and 743 mm in width and length, respectively. Dried ferns are placed transversely on the front of the outer surface of the mummy (Figure 1).

### 3.2. CT study of the mummy

**FIGURE 2:**
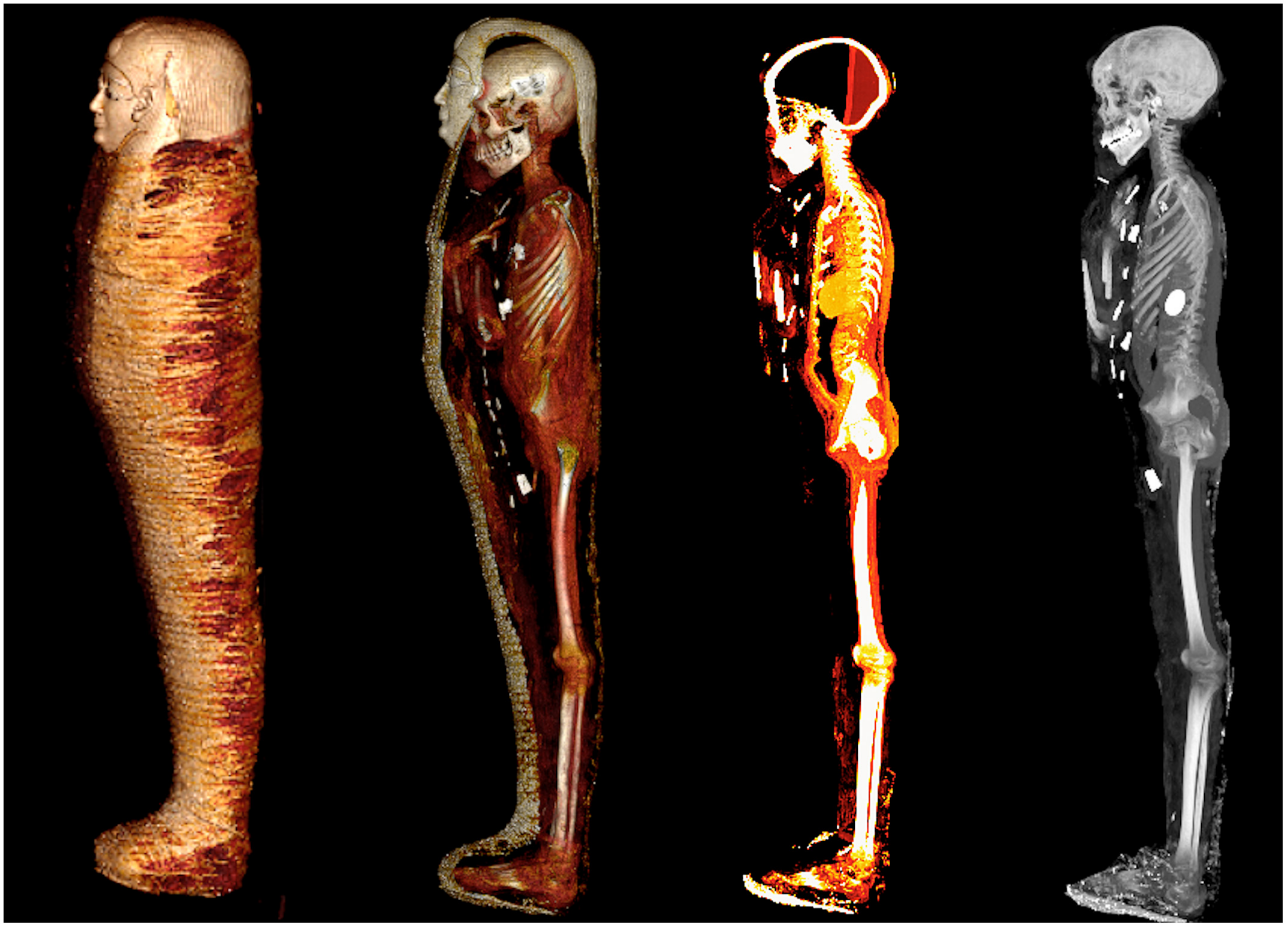
Digital unwrapping visualizations using computed tomography (CT) of the mummy TR 21/11/16/16 at the Cairo Egyptian Museum. A series of four three-dimensional (3D) CT images of the mummy in the left lateral position show the steps of the virtual peeling of the bandages from left to right: the outer surface of the wrapped mummy (far left); partial unwrapping reveals the mummy within the bandages; the totally unwrapped mummy is viewed with a CT window level that highlights the soft tissues and embalming materials; the totally unwrapped mummy is viewed with a CT window level that shows the skeleton and dense amulets related to the mummy (far right).

**FIGURE 3:**
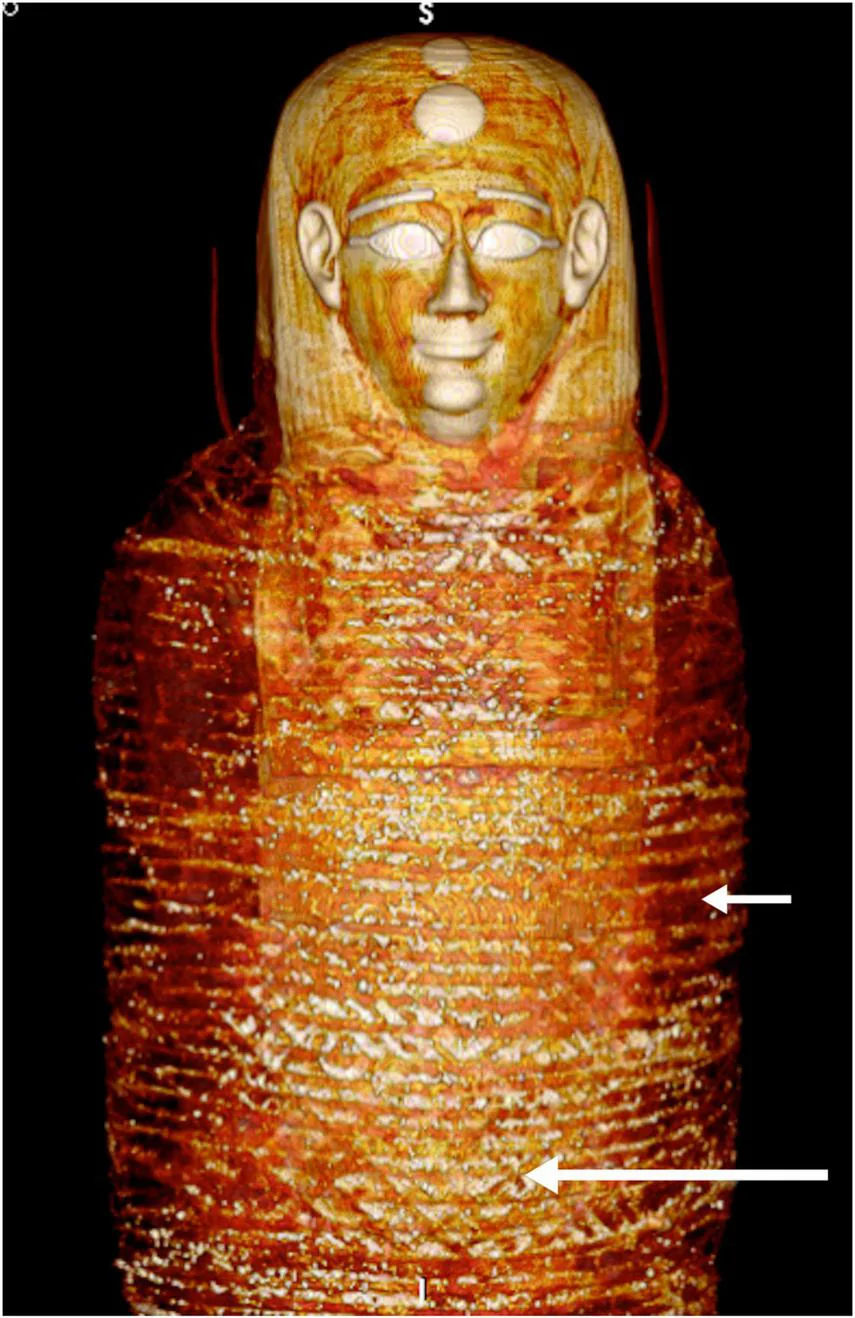
Three-dimensional computed tomography (CT) image of the front of the head and torso of mummy TR 21/11/16 at the Cairo Egyptian Museum showing the head mask and the most outer bandages arranged transversely (short arrow) and diagonally in a criss-cross orientation (long arrow).

We digitally removed the linen layers wrapping the mummy to expose its surface and visualize its interior (Figure 2). CT images show the mummy completely wrapped in several layers of bandages arranged transversely and diagonally in a crisscross orientation (Figure 3). The maximum thickness of the wrappings is 115 mm anteriorly and 43 mm posteriorly. Each of the four limbs is being separately wrapped. The CT density of the most outer layer of the mummy wrappings measures 390–590 HU. The inner linen layers directly wrapping the mummy have a low CT density (-700 HU) alternating with denser layers measuring (30–100 HU) corresponding to linen impregnated with resin.

#### 3.2.1. Preservation status and posture

**FIGURE 4:**
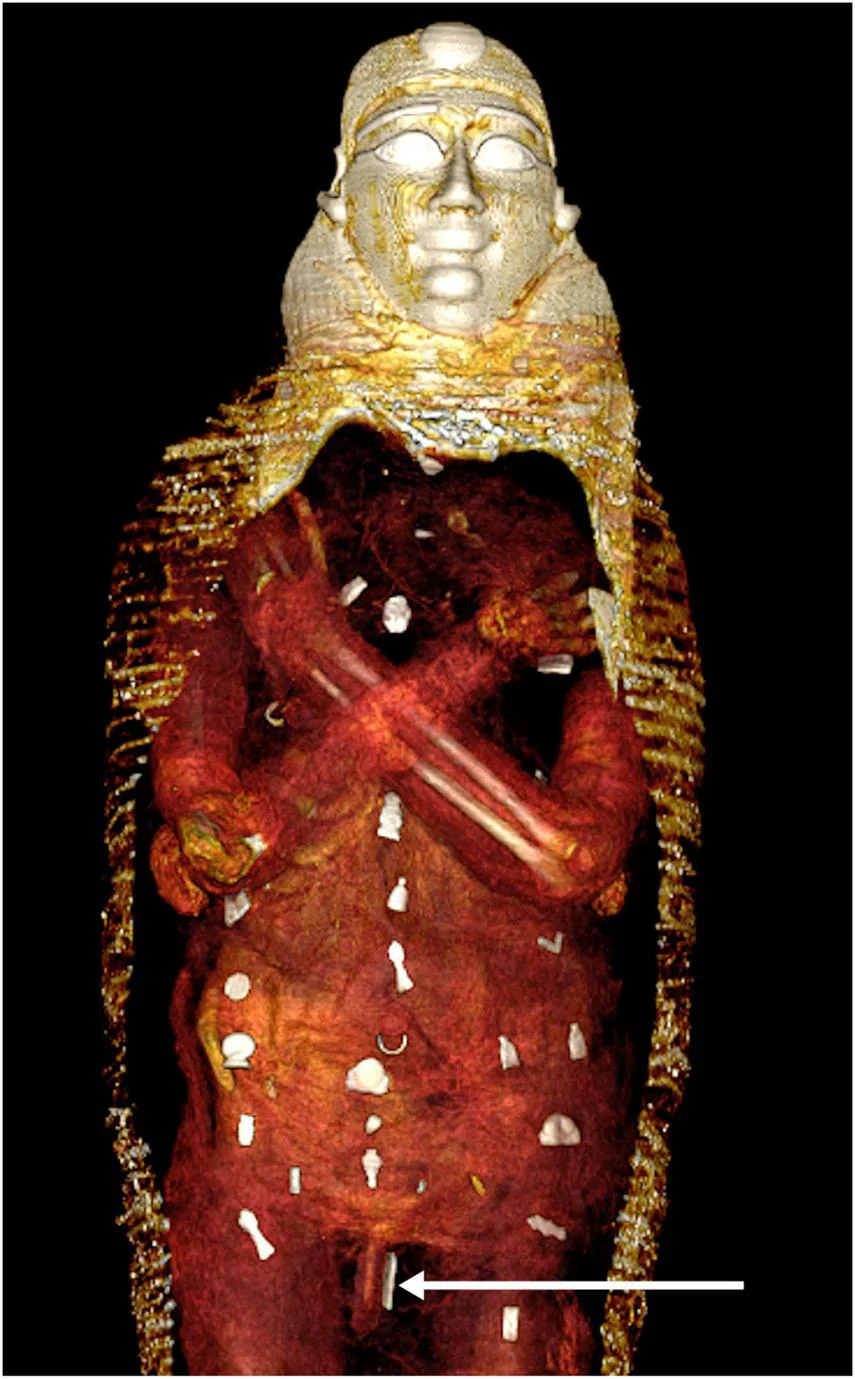
Three-dimensional computed tomography (CT) image of the front of the digitally unwrapped torso of mummy TR 21/11/16 at the Cairo Egyptian Museum shows the crossed arms position and the amulets. The separately wrapped upper limbs are bent at the elbows and crossed in front of the chest. Amulets are seen arranged in three columns on the front surface of the torso. An amulet is placed beside the penis.

The mummy is complete and well-preserved. The body is fully extended with the arms crossed on the front of the chest, the right forearm over the left (Figure 4). The palms of the hands are flat. The neck is slightly flexed with the head tilted down. Otherwise, an intact articulated skeleton with overlying thin desiccated soft tissues.

#### 3.2.2. Stature and long bone measurements

The vertex to heel length measures 1,278 mm. The right and left femur lengths measure 356 and 355 mm, respectively. The right and left tibial lengths measure 285 and 284 mm, respectively. The right fibular and left fibular lengths measure 276 and 274 mm, respectively. The right humeral and left humeral lengths measure 241 and 245 mm, respectively.

#### 3.2.3. Sex

Two-dimensional and three-dimensional CT images of the pelvic regions showed a well-preserved penis measuring 41 mm in length wrapped in a linen sheath (Figure 4 and Supplementary Figure 4).

#### 3.2.4. Age at death

**FIGURE 5:**
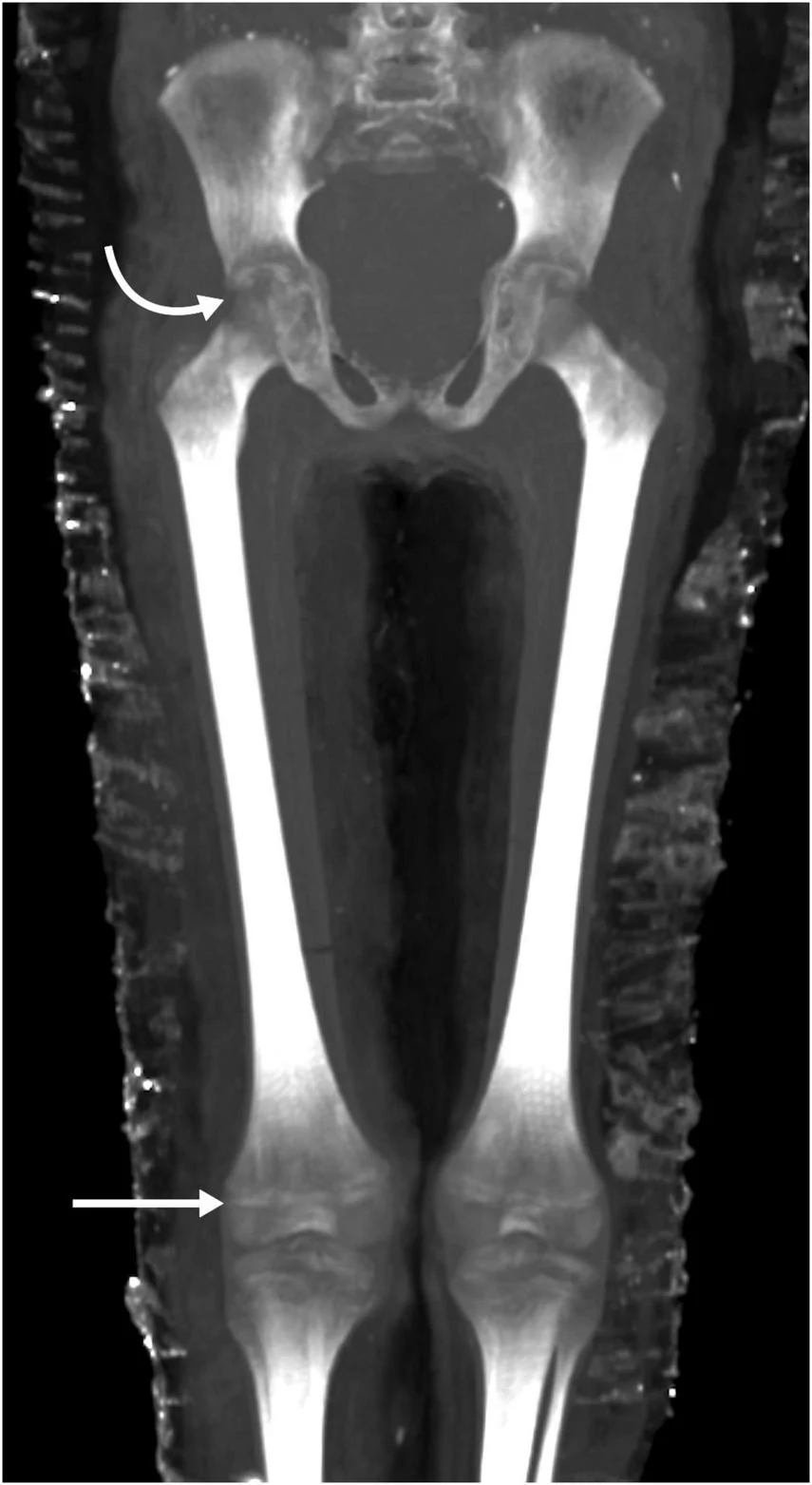
Thick-slab maximum intensity projection (MIP) coronal reconstruction computed tomography (CT) image of the hips and knees of mummy TR 21/11/16 at the Cairo Egyptian Museum shows non-fusion of the epiphyses. Continuous radiolucent gaps separate the epiphysis and diaphysis at the hip joint (curved arrow) and the knee joint (straight arrow) indicating a juvenile person.

The estimated age of the deceased at death was 14–15 years based on the CT examination of the epiphyseal union. The epiphyses around the elbows are closed. The epiphyses remained unfused in the following bones: around the ankles, proximal femur, around the knees, and proximal humerus (Figure 5). The estimated age based on tooth eruption confirmed the bony age. All permanent teeth had erupted except the four third molar teeth, indicating an age older than 12 years and younger than 18 years (10).

#### 3.2.5. Facial features, health, diseases, and cause of death

**FIGURE 6:**
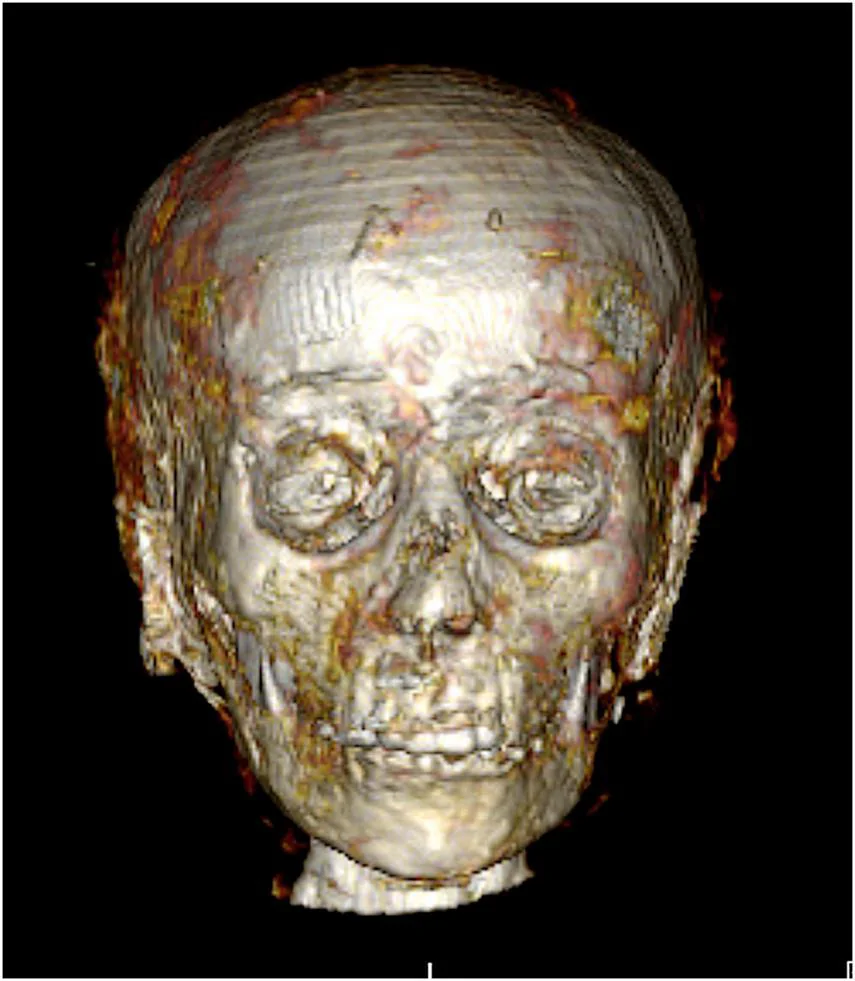
Three-dimensional computed tomography (CT) image in frontal view of the face of mummy TR 21/11/16 at the Cairo Egyptian Museum. An oval face, with a small nose, a narrow chin, and partly open eyes.

-The mummy has an oval face with a small nose, a narrow chin, and partly opened eyes (Figure 6).

**FIGURE 7:**
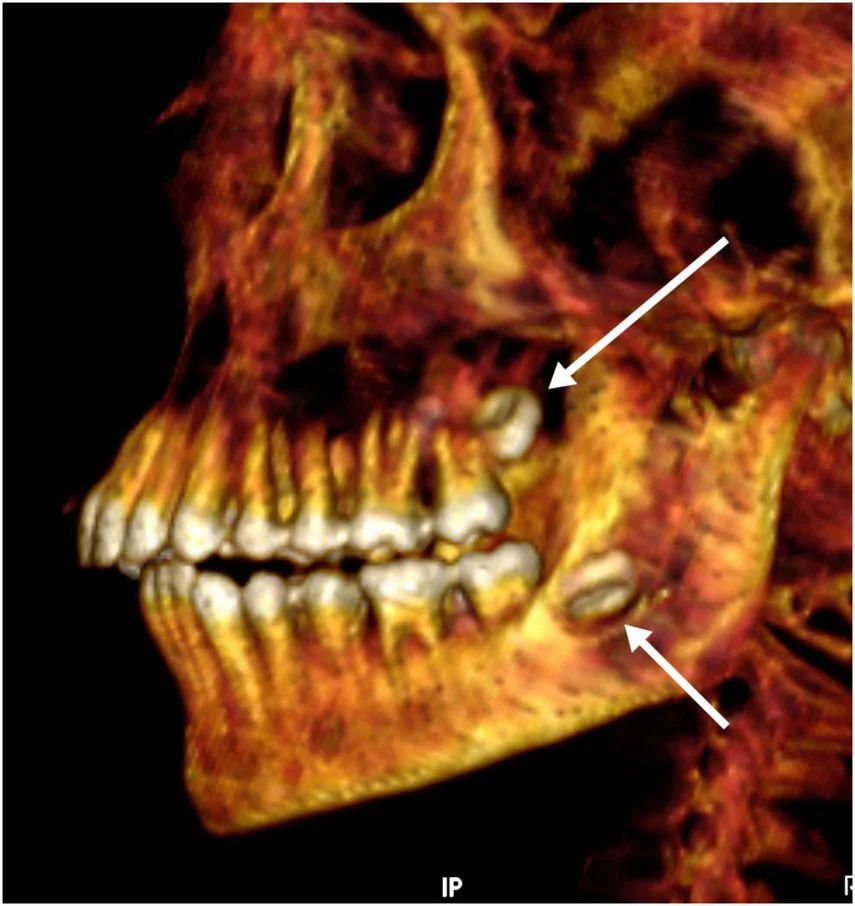
Three-dimensional computed tomography (CT) image of the teeth of mummy TR 21/11/16 at the Cairo Egyptian Museum in left lateral view. A mild overbite and non-erupted upper (long arrow) and lower (short arrow) third molar teeth.

-Good dental condition of the mummy with no evidence of caries, tooth loss, breaks, abscesses, or periodontal disease. The third molar teeth are non-erupted (Figure 7).

-Sacralized fifth lumbar vertebra. No CT evidence of skeletal anomalies, bony malformation, or growth arrest lines.

The cause of death could not be determined radiographically.

#### 3.2.6. Mummification

**FIGURE 8:**
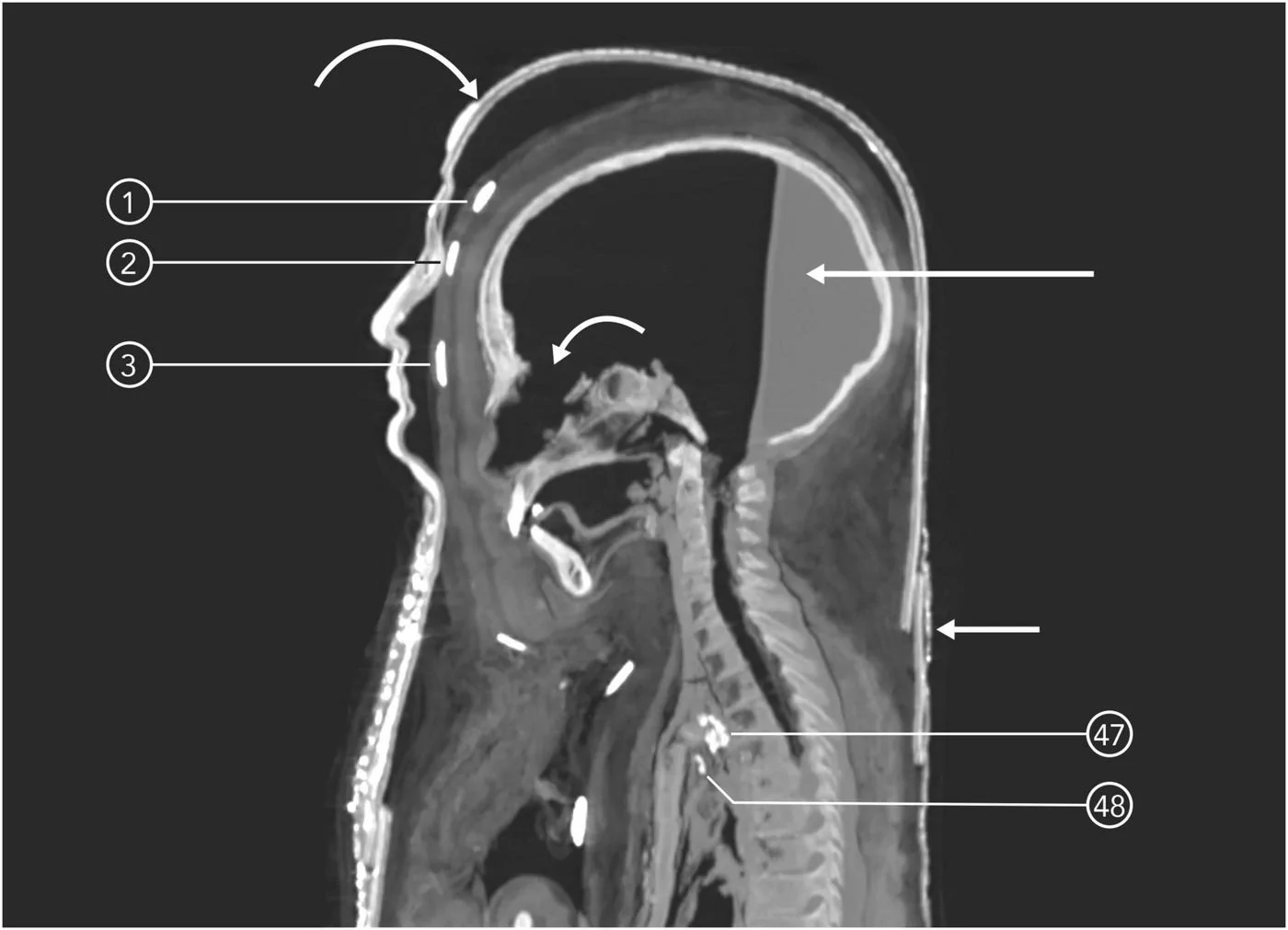
Mid-sagittal computed tomography (CT) image of the wrapped mummy TR 21/11/16’s head at the Cairo Egyptian Museum shows the skull, bandages, and mask. Evidence of brain removal as part of the mummification through a skull base defect (short curved arrow) and resin level at the back of the emptied skull (long straight arrow). The head cartonnage mask is formed of multiple layers (long curved arrow) and shows a fracture at its lower back (short straight arrow). Three dense gold scarab amulets are placed on the midline of the surface of the wrapped head beneath the mask; 1: on the vertex; 2: on the forehead; 3: on the glabella. Two quartz/faience amulets ae seen inside the upper chest cavity: 47: Udjat and 48: Right angle amulet.

-Cranial mummification: The brain has been completely removed (excerebrated) trans-nasally through a large postmortem defect in the anterior skull base (cribriform plate) measuring 37.5 × 23 mm in anteroposterior and transverse dimensions, respectively. A solidified resin with a CT density of 70 HU occupies the posterior one third of the cranial cavity forming a fluid level (Figure 8).

**FIGURE 9:**
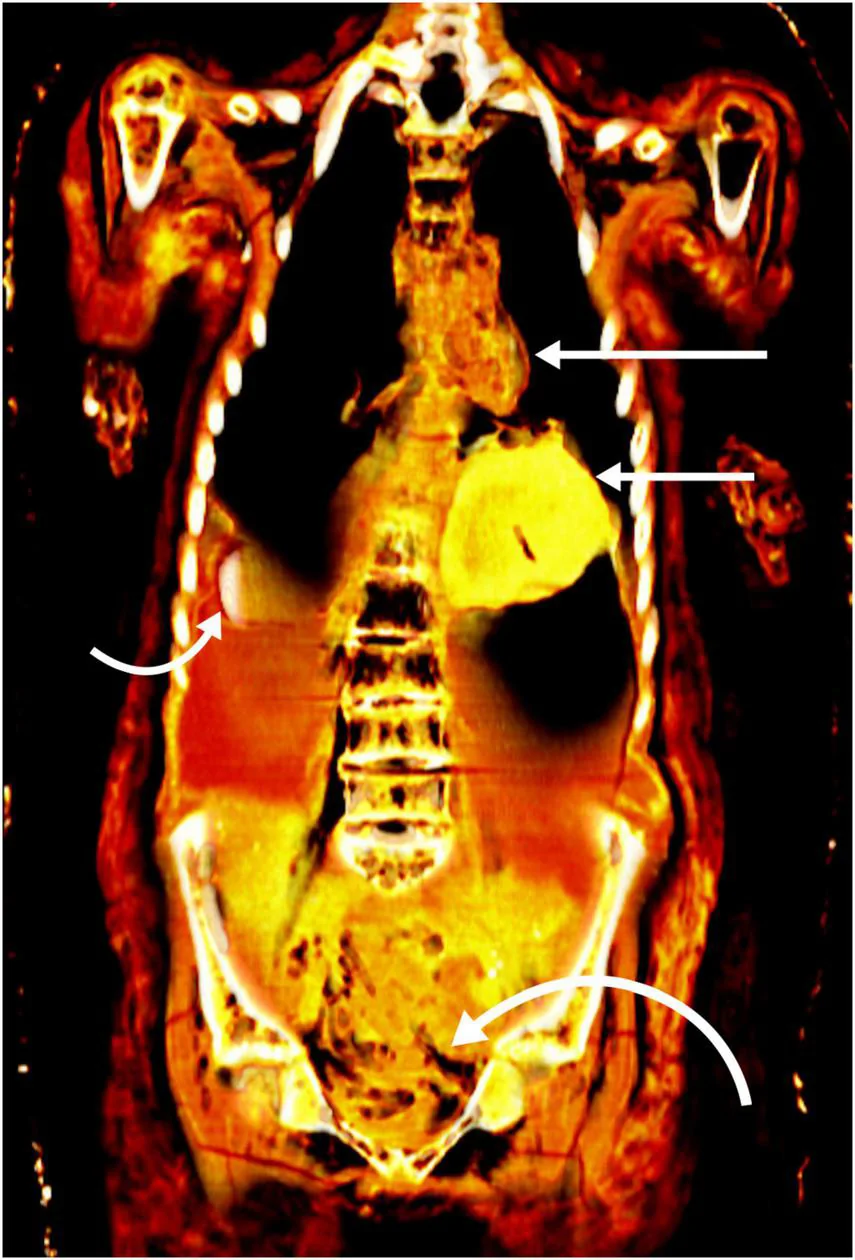
Coronal computed tomography (CT) image of the eviscerated torso of mummy TR 21/11/16 at the Cairo Egyptian Museum filled with pack (short straight arrow), resin with a scarab amulet partially embedded in it (short curved arrow), and loose linen (long curved arrow). Note that the heart was not removed during the mummification (long straight arrow).

**FIGURE 10:**
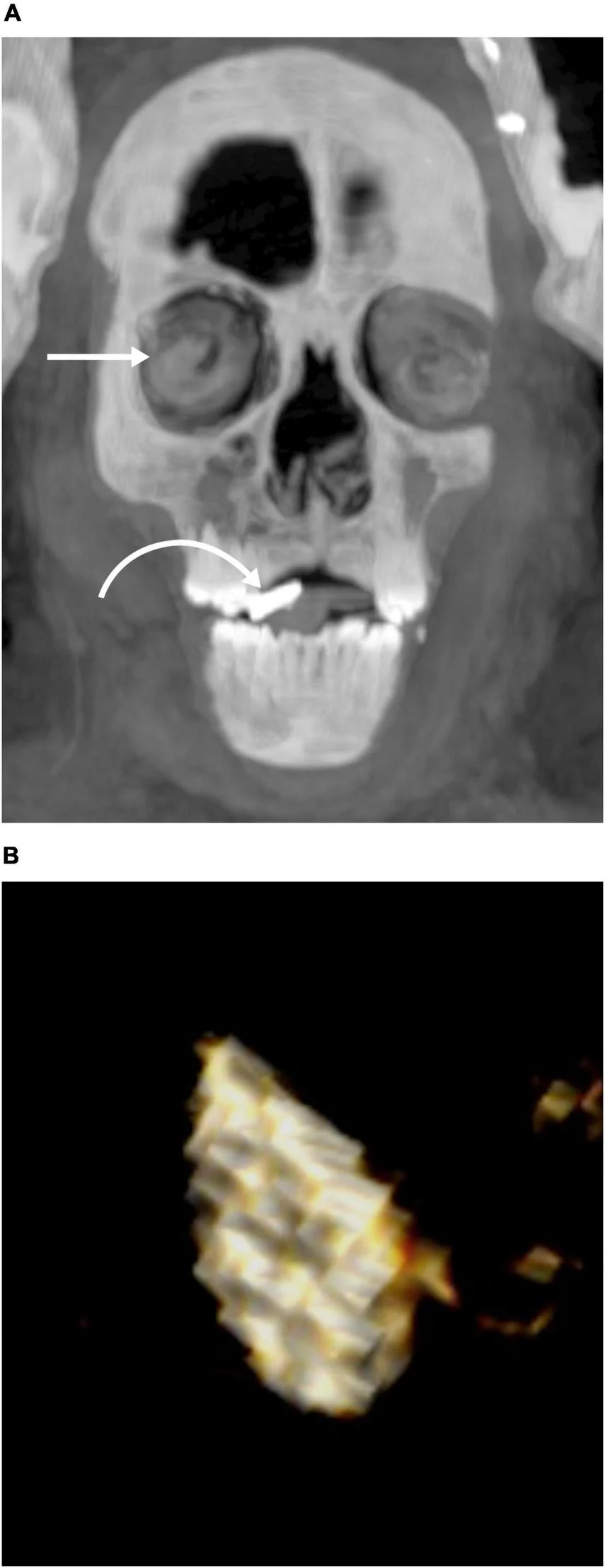
Computed tomography (CT) images of the gold tongue amulet in the mouth of mummy TR 21/11/16 at the Cairo Egyptian Museum. **(A)** Thick slab coronal CT image of the mummy’s head shows a dense (gold) flat amulet inside the mouth (curved arrow). Note that the orbit is packed with linen (straight arrow). **(B)** Three-dimensional CT image of the amulet shows it has flat tongue like shape.

-Body mummification: The viscera were completely removed (eviscerated), except for the heart, which is seen inside the middle chest cavity on the left side. The embalming incision appears in the left lower abdomen; it measures 85 × 62 mm in length and breadth, respectively, and is covered with linen. A well-formed visceral pack is seen inside the upper left side of the body cavity. The pack measures 76 × 55 mm in anteroposterior and transverse dimensions, respectively and has compact contents (likely a visceral pack). Loose textile bundles are seen within the pelvic cavity. Structureless resin occupies the abdominal cavity (Figure 9). Loose textile bundles are placed inside both orbits (Figure 10). No subcutaneous packing could be detected in the face or the body of the mummy.

#### 3.2.7. Amulets related to the mummy

The CT images show a total number of 49 amulets related to the mummy (Table 1). Three amulets are placed inside the torso cavity (Figures 8, 10), one amulet inside the mouth (Figure 10), and 45 amulets are placed on the body surface or in between its wrappings (Figure 4).

**TABLE 1:** Computed tomography (CT) findings of amulets related to the mummy.

#	Amulet location in relation to the mummy	Material, amulet identity, and measurements (length, breadth, width mm; CT density HU)
1	Head: on vertex midline	A gold scarab (15.5 × 9.4 × 5; 3,071)
2	Head: on forehead midline	A gold scarab (18.3 × 20 × 5; 3,071)
3	Head: on glabella	A gold scarab (18.7 × 16 × 4.6; 3,071)
4	Head: inside the mouth	A gold tongue (16 × 8.6 × 5.7; 3,071)
5	Front of neck (C7 level)	A gold flask (19.4 × 8 × 3; 3,071)
6	Front of torso: midline (D1 level)	A gold Djed (30 × 11.3 × 5.6; 3,068)
7	Front of torso: right (D6 level)	A gold Udjat (25.5 × 19.5 × 16.2; 3,069)
8	Front of torso: right para-midline (D6 level)	A gold Double-falcon-plume (18 × 8.3 × 4.5; 3,070)
9	Front of torso: midline (D6 level)	A gold scarab (21 × 15 × 5; 3,069)
10	Front of torso: left (D6 level)	A gold Udjat (22 × 21 × 5.6; 3,065)
11	Front of torso: left wall and arm (D6 level)	A stone Udjat (27 × 24 × 5.2; 2,228)
12	Front of torso: right lateral (D9 level)	A quartz/faience crescent (15 × 5 × 2; 1,594)
13	Front of torso: midline (D9-10 level)	A gold scarab (16 × 11 × 5.7; 3,070)
14	Front of torso: to left (D9-10 level)	A quartz/faience crescent (18 × 3 × 2.7; 2,027)
15	Front of torso: right (L1 level)	A stone serpent head (19.9 × 9.8 × 2.5; 2,500)
16	Front of torso: midline (L1 level)	A gold Tyt (28 × 11 × 5; 3,069)
17	Front of toros: left lateral (L1 level)	A gold plate/palette (20 × 12 × 5; 3,071)
18	Front of torso: left lateral between body surface and left arm (L1 level)	A quartz/faience Udjat (21 × 18.5 × 4.6; 2,300)
19	Front of torso: left lateral between body and left arm (L2 level)	A quartz/faience scarab (18 × 14 × 4; 1,300)
20	Front of torso: right lateral (L3 level)	A gold Double-ostrich-plume (24 × 10 × 5; 3,071)
21	Front of torso: midline (L3 level)	A gold flask/bottle (18 × 8 × 4.6; 3,068)
22	Front of torso: right (L4 level)	A stone placenta (15 × 14.6 × 3; 2,509)
23	Front of torso: midline (L4 level)	A gold Djed (26 × 8 × 3; 3,071)
24	Front of torso: left lateral (L4 level)	A gold right angle (15 × 13 × 4; 3,070)
25	Front of torso: right lateral (S1 level)	A gold Nou flask (18.7 × 18 × 6.8; 3,069)
26	Front of torso: midline (S1 level)	A quartz/faience crescent (16 × 15.8 × 6; 1,700)
27	Front of torso: left lateral (S1 level)	A gold flask/bottle (16 × 10 × 4; 3,071)
28	Front of torso: further left lateral (S1 level)	A gold flask/bottle (18 × 10 × 6; 3,071)
29	Front of torso: right lateral (S3 level)	A gold Ajet (23 × 17 × 5.5; 3,070)
30	Front of torso: right lateral (S4 level)	A gold cylinder (14 × 7 × 7; 3,070)
31	Front of torso: midline (S4 level)	A gold bead (10 × 6.8 × 6; 3,070)
32	Front of torso: left lateral (S4 level)	A quartz/faience Ajet (22 × 16.5 × 7.7; 2,138)
33	Front of torso: right lateral (coccyx level)	A gold cylinder (13.8 × 13.8 × 5.7; 3,070)
34	Front of torso: midline (coccyx level)	A gold flask (20 × 5.7 × 10; 3,071)
35	Front of torso: left lateral (coccyx level)	A quartz/faience pyramid (10 × 6 × 5; 1,914)
36	Front of torso: left lateral (coccyx level)	A fired clay hollow cylinder (12 × 6 × 6; 648)
37	Front of right upper thigh	A gold Djed (30 × 8.5 × 4; 3,071)
38	Front of left upper thigh	A gold zig zag line of water (23 × 8.5 × 4.5; 3,070)
39	In between upper thighs: at the left side of the penis	A gold two-finger (30 × 16.4 × 6.5; 3,071)
40	Font of left upper thigh	A gold plate/palette (18 × 8.5 × 3.5; 3,070)
41	Back of left upper thigh	A fired clay triangular amulet (10.2 × 8.2 × 2.4; 550)
42	Back of left upper thing	A fired clay plate/palette (11.3 × 8.7 × 3.5; 564)
43	In between both lower thighs (knee level)	A quartz faience Udjat (9.4 × 8.2 × 3.5; 1,744)
44	In between both upper legs	A quartz/faience Udjat (17 × 9 × 3.8; 1,700)
45	In front of middle left leg	A fired clay Udjat (18 × 15 × 3.6; 1,100)
46	In front of lower left leg	A quartz/faience Udjat (15 × 8.3 × 3.7; 1,731)
47	Inside torso cavity (D4 level)	A quartz/faience Udjat (18 × 13 × 12; 2,016)
48	Inside torso cavity (D5 level)	A quartz/faience Right-angle (8.6 × 3.1 × 2.7; 1,692)
49	Inside torso cavity right lateral (D12 level)	A gold disk (likely a heart scarab) (38.5 × 28 × 9; 3,027)

**FIGURE 11:**
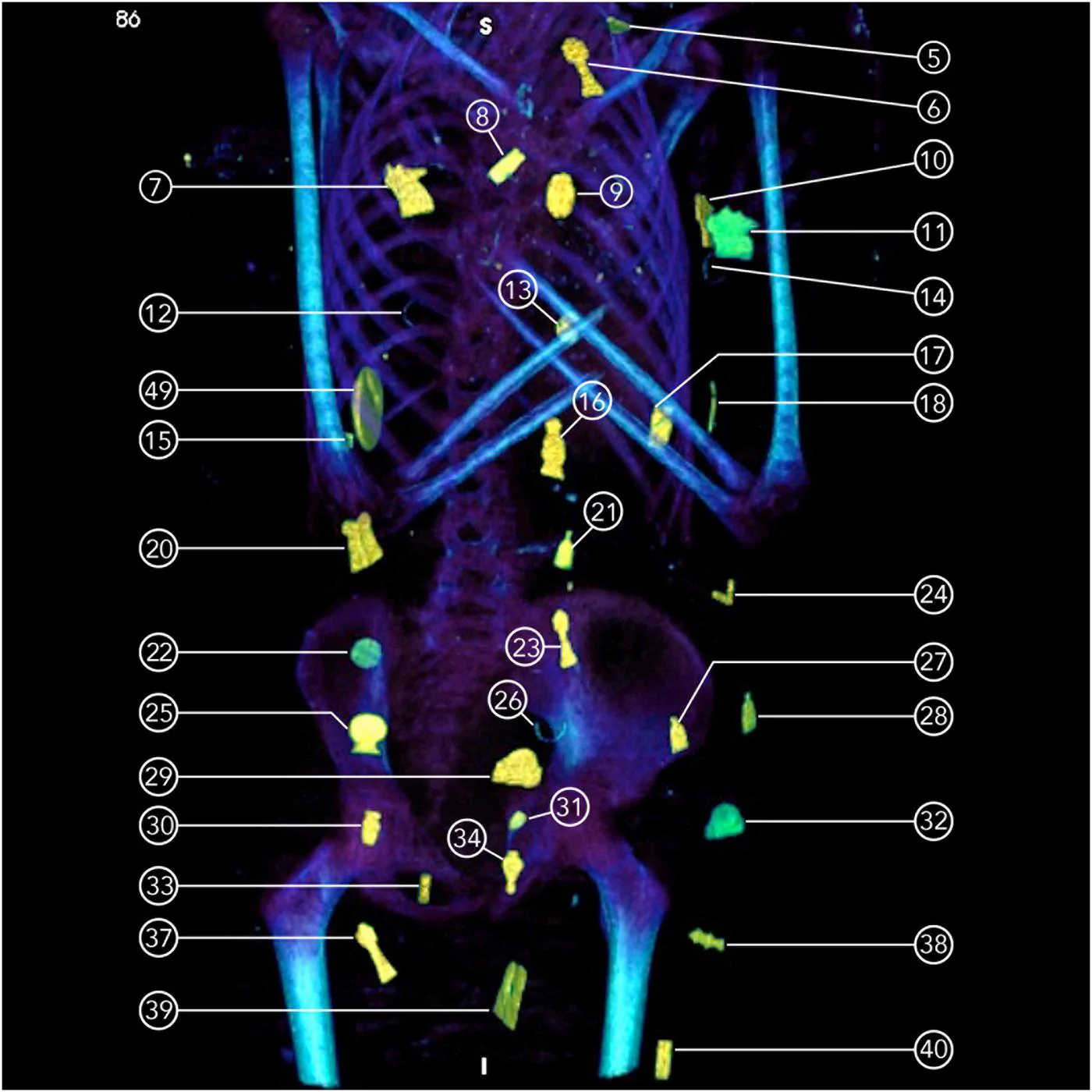
Thick slab coronal computed tomography (CT) image of the torso of mummy TR 21/11/16 at the Cairo Egyptian Museum showing amulets placed on the mummy’s surface and between the wrappings (5: gold flask; 6: gold Djed; 7: gold Udjat, 8: gold Double-falcon-plume; 9: gold scarab; 10: gold Udjat; 11: stone Udjat; 12: quartz crescent; 13: gold scarab; 14: quartz crescent; 15: stone serpent head; 16: gold Tyt; 17: gold plate; 18: quartz udjat; 20: gold double ostrich plume; 21: gold flask; 22: stone placenta; 23: gold djed; 24: gold Right angle; 25 gold Nou flask; 26: quartz crescent, 27: gold flask, 28: gold flask, 29: gold ajet; 30: gold cylinder; 31: gold bead; 32: quartz Ajet; 33: gold cylinder; 34; gold flask; 37: gold Djed; 38: gold zigzag; 39: Two-finger; 40: gold plate).

The distribution of the 45 amulets on the mummy surface/wrappings is: four on the head and neck region (Figure 8); 31 amulets on the torso (Figures 4, 11), and 10 amulets on the lower limbs.

Computed tomography images identify the amulets according to their morphology as follows: Udjat (*n* = 9), Scarab/Scaraboid (*n* = 7); Flask/bottle (*n* = 5); Djed pillar (*n* = 3); Crescent (*n* = 3); Cylinder amulets (*n* = 3); Plate/Palette (*n* = 3); Ajet (*n* = 2); Right-angel (*n* = 2); and one of each of the following: a Serpent-Head, an Isis-Knot (Tyt), a Double-Falcon-Plume, a Double-Ostrich-Plume, a Placenta, a Zig zag line of water, a Pyramid, a Triangle, a bead, a Nou flask, and a Tongue amulet inside the mouth (Figure 10). A Two-finger amulet is seen placed at the left side of the wrapped penis (Figures 4, 11); the 3D CT image reveals the details of the nails (Supplementary Figure 4).

In several locations, we note a symmetrical balanced pattern in the horizontal arrangement of the amulets on the surface of the mummy’s torso. Udjat amulets are placed in the right column (amulet #7) and the left column (amulets #10, 11) at the same level in the mid thorax. Ajet amulets are located at the mid pelvic level in the right column (amulet #30) and in the left column (amulet #33).

Based on their CT densities, the materials the amulets are made of are metal (likely gold) (*n* = 30); quartz/faience (*n* = 12); semiprecious stones (*n* = 3); and fired clay (*n* = 4).

The length of the amulets ranges between 8.6 and 38.5 mm with an average of 19.2 SD 5.99 mm. The breadth of the amulets ranges between 3–28 mm with an average 11.8 mm SD 5.34; and the width ranges between 2–6.2 mm with an average 5.09 mm SD 2.42. The largest amulet is the scarab placed inside the mummy’s torso cavity with a maximum length of 38.5 mm (Figures 9, 11, and Supplementary Figure 5).

**FIGURE 12:**
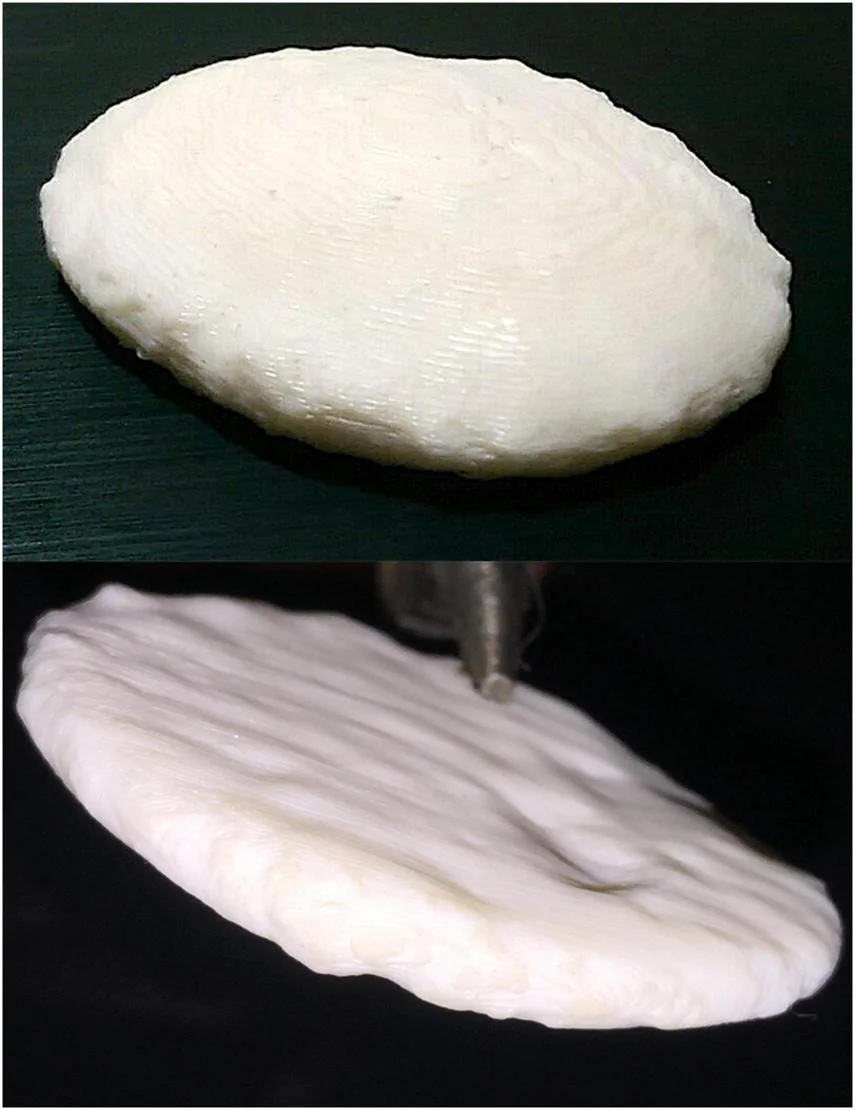
Picture of the front and back of the three-dimensional (3D) printed heart scarab amulet inside the torso cavity of mummy TR 21/11/16 at (Cairo Egyptian Museum). The amulet is made of plastic and has a discoid shape with a convex surface (at the top of the picture) and a flat surface (at the bottom of the picture) with depression marks likely representing inscriptions.

We 3D printed the largest amulet, the scarab inside the mummy’s torso in its real size and documented the CT findings of the amulet. Tactile and visual examination of the printed amulet revealed a discoid object with a convex side and an opposing flat surface with engraved marks likely representing inscriptions (Figure 12).

#### 3.2.8. Mask and external ornamentation

Additional funerary accessories were placed on the surface of the wrapped mummy in the form of a gilded head mask (covers the head and shoulders), a pectoral cartonnage (covers the front of the torso), and a pair of sandals.

The mask covers circumferentially the head of the wrapped mummy and extends in a tripartite wig configuration beneath the wrappings to cover the front and the back of the mummy’s shoulders (Figures 2–4). The height of the mask measures 349 mm in front and 297 at the back; 183 mm in width, and 228 mm in depth. The mask is molded to show idealized human facial features. The mask is formed of a low-density inner layer of linen or papyrus (30 HU) covered by a thin layer of a higher density material corresponding to the gilded part (1,890–1,930 HU) (Figure 8). The average thickness of the mask is 6 mm. The maximum thickness of the gilded layer measures 6–8 mm at the nose, and chin, and a circular ornament in the middle of the forehead measures 34.1 mm in diameter. Eyebrows and widely opened eyes are made of separate pieces of high CT density material mounted on the front of the cartonnage. Each eyebrow piece measures about 49 × 3.8 mm, and the eye piece measures 51 × 1.79 mm at its maximum transverse and height. Two stone disks are mounted at the center of the mask’s vertex and below the chin measuring 32.6 and 25.4 mm in long axis, respectively (Figure 3). The lower back of the mask shows a complete transverse fracture (Figure 8).

A rectangular sheet of cartonnage (pectoral sheet) is placed in the center of the front of the torso surface, overlapping with the lower end of the head mask. The pectoral cartonnage sheet is covered by the outer wrappings. It measures 237 mm in length. The upper end of the sheet is wider (192 mm) than its lower part (135 mm). The thickness of the sheet is 15.5 mm, formed of a low-CT density linen/papyrus (18–30 HU).

**FIGURE 13:**
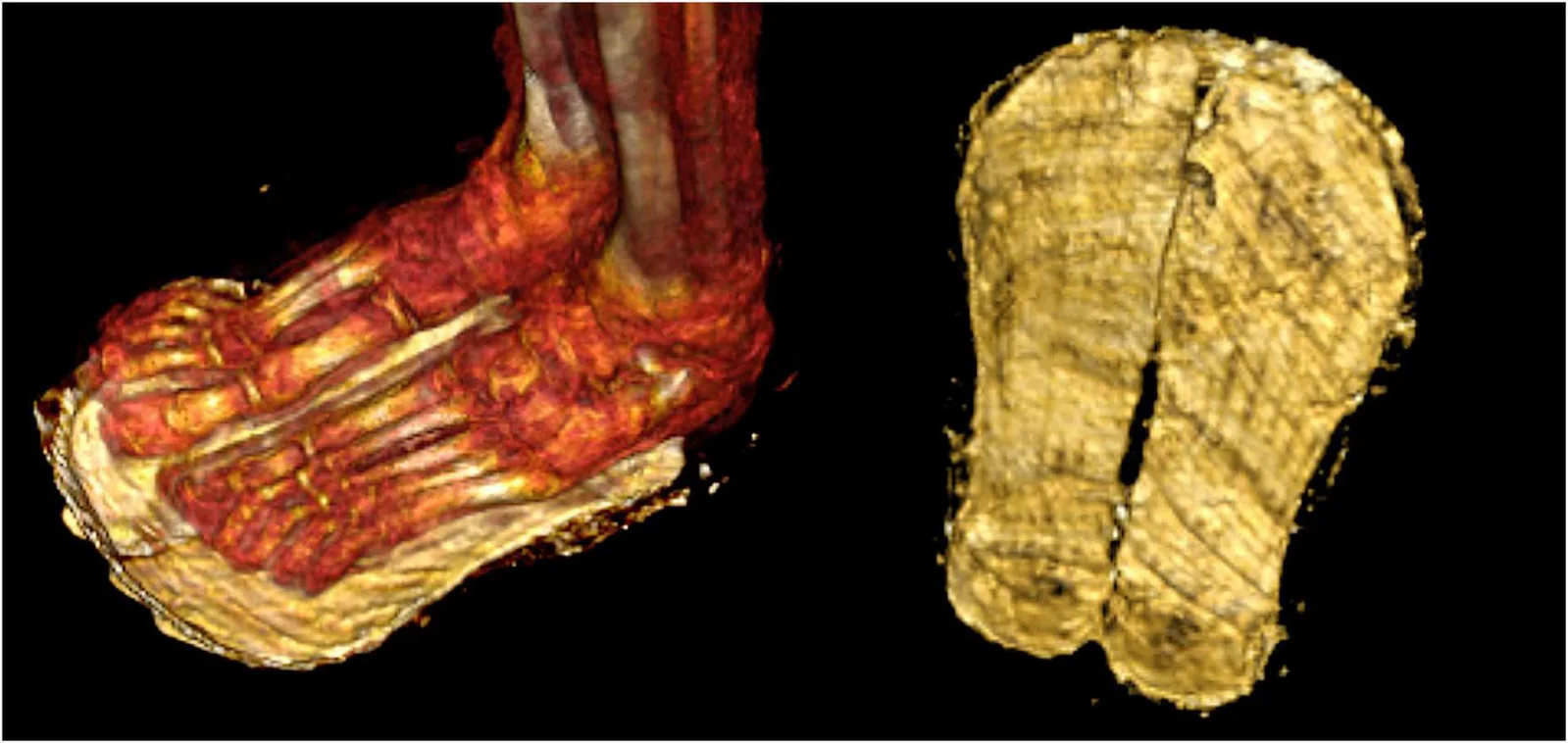
Three-dimensional (3D) computed tomography (CT) image of the sandal of mummy TR 21/11/16 from the Cairo Egyptian Museum. A pair of open sandals without strips (on the right side) are placed on the soles of the mummy’s feet (on the left side).

Sandals: A pair of sandals are placed on the soles of both feet. The sandal is an open shoe without strips, straight without divergences or constrictions, and has a rounded heel (Figure 13). The measurements of the sandals are: the right one measures 178.5 × 60 mm in length and breadth; the left one measures 174.2 × 75.2 mm in length and width, respectively. The CT density of the sandal measure (452–590 HU) is likely made of treated papyrus or palm fibers.

The lower part of the head mask and the pectoral cartonnage are covered by outer wrapping layers made of plastered linen.

## 4. Discussion

In this report, we investigated a fully wrapped mummy with a golden mask that had been stored in the basement of the Cairo Egyptian Museum for over a century. The mummy came from Nag el-Hassaya, a cemetery from the Ptolemaic period (305–30 BC) in Edfu, about 800 km south of Cairo. The mummy was discovered inside two coffins: an inner sarcophagus wooden coffin with an anthropoid shape from the late Ptolemaic era; and an outer coffin with inscriptions in Greek letters. The mummy had never been examined before, and there was no additional information about the mummy’s identity in the museum records.

Mummy digital unwrapping using CT has replaced invasive physical examination and dissection in the past. CT digital unwrapping offers non-invasive results about the mummy’s age, sex, health issues, as well as mummification style (6). The CT images in this study show a well-preserved male mummy with a preserved penis. In the sagittal reconstructed CT image, the mummy is for a juvenile with a height of 127.7 cm. A CT scan can accurately estimate the age of a subadult mummy by detecting developmental changes such as erupted teeth and epiphyseal closure (5, 17). CT images show that all permanent teeth have erupted except the third molar teeth, indicating a death age between 12 and 18 years. The skeleton epiphyseal union of the mummy estimated the age at death to be 14–15 years.

The mummy’s dental condition was excellent with no signs of tooth loss, breaks, abscesses, periodontal disease, or caries. Previous research found that tooth abscesses and periodontal diseases were common in ancient Egyptian human remains. Previous research studies suggested that ancient Egyptians had worn teeth that increased with the individual’s age (18, 19). The absence of dental disease in this young person cannot be used to generalize the ancient Egyptians’ knowledge of hygiene and dentistry; more research with a larger sample size is required. Several ancient Egyptian mummies in good dental condition have been discovered in recent studies (5, 6). In ancient Egypt, hygiene was important; people washed everyday and used perfumes and cosmetics. Oral hygiene regimens appeared to be important in ancient Egypt. Hesy-Ra was an Egyptian scribe who lived around 2,600 BC and is regarded as the first dental practitioner on the written record (20). Recipes for mouthwashes, toothpaste, and pharmaceutical preparations for the treatment of dental conditions were found in ancient Egyptian medical papyri (21). More research is needed to gain a better understanding of dental health in ancient Egypt. The CT examination of the mummy under study reveals no skeletal diseases, bone abnormalities, or growth arrest lines. When a juvenile experiences episodes of food scarcity or malnutrition, dense lines appear in the bones, indicating a growth disturbance (22). Growth arrest lines were discovered in 85% of the 21 mummified ancient Egyptian children housed at European museums (23). Although there were no growth arrest lines on the juvenile mummy in this study, this does not rule out the possibility that he was malnourished.

There was no evidence of trauma or disease that leaves bone markers. The cause of death could not be determined.

In ancient Egypt the family was important. Children were valued and well-taken care of. A child’s death in a family must be mourned and preserved for the afterlife through proper ritual treatment and mummification (13).

### 4.1. Mummification style

The CT images in this study show the body’s posture of the mummy beneath the wrappings. The body is fully extended with the arms crossed in front of the chest. In ancient Egypt, arm positioning changed over time. The crossed arm posture over the chest has been linked to royal mummies dating back to the New Kingdom (18th–20th Dynasties), beginning with King Amenhotep I (18th Dynasty c.1525-1504 BC) (5, 6). Non-royal mummies from the New Kingdom usually had their arms extended. Although the crossed arm posture was associated with kingship in the New Kingdom, it was later widely applied to the population. Crossed arms first appeared in non-royal mummies during the 22nd and 23rd dynasties and continued through the Late Period, and Greco-Roman era (24).

According to their religion, mummification was practiced in ancient Egypt to preserve the body after death for a perfect afterlife (1). The quality of mummification varied according to the person’s socio-economic status. According to Herodotus, the highest levels of the mummification process included excerebration and evisceration (3). In the studied mummy, the embalmers removed the brain and viscera except the heart and stuffed the emptied body cavities with expensive embalming materials, resin, and linen packs. The heart was left in place because it represented an important spiritual symbol to the ancient Egyptians. Early in the New Kingdom, brain removal became a common procedure in mummification, peaking during the Ptolemaic Period (25).

### 4.2. Wrapping and amulets

Wrapping the mummified body in linen bandages was an important step in the ancient Egyptian mummification process (3). Beginning in the New Kingdom, mummy wrapping became more sophisticated as embalmers wrapped each limb individually, followed by the entire body (6). CT images show that the mummy in this study has been completely wrapped, for each of the four limbs wrapped separately. Strips of linen wrapped transversely and crisscrossed across the surface of the body from neck to toes, a common wrapping style in ancient Egypt’s Greco-Roman eras. The findings point to an elaborate wrapping process as part of a high-quality mummification process.

The mummification process in ancient Egypt included reciting spells and placing charms and amulets inside the mummy and between its wrappings to protect the deceased from dangers during the journey to the afterlife (3). CT scans revealed amulets in the mummies of Kings such as Amenhotep I, Seti I, Ramesses II, and Ramesses III (5, 6). Amulets were available in a variety of sizes, shapes, and functions; some represented a symbol, a goddess or a god (26, 27). In this study, digital unwrapping of the mummy revealed 49 amulets presented in 21 different shapes (Table 1 and Supplementary Table 1).

The ancient Egyptians believed that the magical power of an amulet was derived from its material, shape, and color (27). Amulets in ancient Egypt were frequently made of various materials, such as expensive gold and semiprecious stones or inexpensive fired clay (6). The use of expensive materials to make amulets in the mummy was most likely to ensure the deceased safe journey to the life after (27). However, the cost of materials for amulets placed during mummification was determined by the deceased’s socio-economic status (1). CT can help determine the material of an amulet by measuring its density (14). Thirty amulets in the studied mummy were made of metal, accounting of 61% of the total number of amulets. Other amulets investigated were made of semiprecious stones, quartz, faience, or fired clay. The presence of golden amulets within the studied mummy indicated an expensive mummification process.

According to ancient Egyptian belief, the amulets were carefully placed within the mummy to achieve their desired magical effects related to the resurrection of the body for the afterlife (26). Three amulets were discovered inside the mummy’s torso: Udjat, a Scarab, and a Right-angle. The right-angle symbol, a tool which architects use to level construction sites, was thought to bring balance and leveling to the deceased (3, 26).

We discovered a gold flat amulet inside the mummy’s mouth using CT images. The amulet represents the tongue to ensure the deceased speaks in the afterlife, which was a common practice during the Greco-Roman era. Golden tongue amulets were discovered inside the mouths of some mummies found in Taposiris Magna Temple in Alexandria (28). The presence of a golden tongue amulet in the studied mummy corresponds to the suggested dating.

Symmetrical balanced arrangements of amulets in columns were reported in ancient Egyptian mummies (3). Amulets were purposefully arranged on the mummy surface and between the wrappings to create three columns: a midline and bilateral. The horizontal arrangement of the amulets on the surface of the mummy’s torso has a symmetrical pattern in several places. Udjat amulets, for example, were found in the right and left columns of the mid thorax. The Udjat was the most popular amulet used by both the living and the dead in ancient Egypt; it represents the healed eye of the god Horus and represents healing power and regeneration (3). The most identified amulets in this study were Udjat and scarabs.

The scarab amulet, which represents the god Kheper, who rolls the sun across the sky, was thought to resurrection power by the ancient Egyptians (27). The CT images of the studied mummy revealed seven scarab amulets: six on the surface of the head and one inside the torso cavity. The heart-scarab is a large scarab amulet inscribed with sacred text from The Book of the Dead, an ancient Egyptian religious book (26). The amulets in this study ranged in length from 8.6 to 38.5 mm. In this study, the largest amulet was a gold disk with engraved markings on its surface found inside the mummy’s torso cavity likely representing a heart scarab.

The Two-finger amulet has the shape of the index and second fingers of the right hand. The amulet first appeared in the Late Period (664–332 BC). The Two-Finger amulet is commonly found in the lower torso of mummies, likely to protect their embalming incision (27). We found a Two-finger amulet in the CT images of the studied mummy placed beside the penis; the amulet had a very high CT density. Two-Finger amulets were usually made of dense black stones like hematite and sometimes gold (29).

Other amulets found on the surface or between the wrappings of the studied mummy served a variety of functions, including to ensure a safe journey to the afterlife. The Flask or Bottle amulet represents the situla (a ritual metallic bucket used to carry holy water). Djed amulet represents the backbone of god Osiris and ensures the revival of the deceased. The Tyt amulet (Isis knot) invokes Isis’s protective power. The Double-plume amulet represents two lives, the spiritual and material (3, 27).

### 4.3. External ornamentation

Mummified bodies in ancient Egypt were covered with a variety of elements ranging from simple outer bandaging to rigid, highly decorated cartonnage castings (3). Cartonnage was made by gluing layers of linen or papyrus, molding them to the desired shape, and then coloring or gilding them (30). Cartonnage was first used as funerary coffins in the Old Kingdom (2,686–2,181 BC), but it gained popularity later in the Ptolemaic and early Roman Periods (330 BC–250 AD) (3, 30). The cartonnage was sometimes made to envelop the entire body. However, during the Ptolemaic Period, a helmet-mask, in addition to several separate cartonnage elements fixed to the wrapped mummy replaced the full body cartonnage (3). CT images in this study identified two separate pieces of cartonnage: a head mask with facial features and a tripartite wig covering the mummy’s head and shoulders, as well as a piece of pectoral cartonnage covering the middle of the front of the chest. It should be noted that the lower part of the head mask as well as the whole pectoral cartonnage are visually hidden beneath the outer wrappings but are detectable in CT images. A CT examination can thus provide valuable information about the full extent of the cartonnage, its shape, and densities (6, 31, 32). The CT densities of the cartonnage varied according to its material (6). In concordance with previous CT studies of gilded masks dated to Greco-Roman eras, the head mask in the studied mummy has inner linen/papyrus layers with low CT densities and an outer gilded layer with higher CT densities (32, 33). This lavish gilded mask confirms the high socio-economic status of the deceased. The CT study identified a fractured lower back of the head mask; this information helped the museum’s curator understand the vulnerable status of the mask when handling the mummy. CT examination of cartonnage shows its preservation status and may help in its restoration (31, 33).

Ancient Egyptians commonly used footwear to protect their feet. Sandals were worn by royals, priests, and ordinary men, women, and children. The ancient Egyptians were expert shoemakers, creating sandals out of woven palm fibers, papyrus, leather, and gold. Both sides of King Narmer’s palette feature sandals (3,100 BC). Sandals were buried with mummies (34). CT images of the studied mummy revealed an open sandal made of woven fibers under the wrappings. Sandals were probably placed on the mummy to enable the deceased to walk and leave the tomb in the afterlife. According to the ancient Egyptians’ ritual book (The Book of The Dead), the deceased had to wear white sandals to be pious and clean before reciting its verses (26, 34).

Three-dimensional (3D) printing employs a special printer that prints an object’s 3D data using various materials such as plastics, resins, or metals. A 3D surface model of an object can be created using photogrammetry (a camera that takes photos in multiple directions) or CT scans (35). 3D printing has been increasingly used in studying archeological artifacts as it enables the capture of more details of the object (7). We 3D printed the largest amulet, the scarab inside the mummy’s torso, in its real size using plastic material. 3D printing enabled tactile and visual examination of the amulet. The printed discoid amulet showed a high level of detail. The engraved marks on its flat surface likely represented ritual inscriptions to protect the heart. 3D printing, based on a CT scan of the original object, enables replicating archeological artifacts at museums to be used in studies, to enhance the interactive experience of visitors, especially the visually impaired, and to be purchased as souvenirs (7, 35).

The plethora of data provided by the CT examination, promoted the mummy’s transfer from the storeroom to be displayed at the museum’s showroom. We produced a short documentary movie to be played next to the Mummy, including its CT data and images. Holding the 3D printed golden scarab amulet provides a unique interactive experience for the visitors. The mummy has been given a nickname for display: the “Golden Boy” because it was wearing a golden mask and was adorned with thirty golden amulets. The display’s goal was to humanize this individual from the past to teach modern people about life in ancient times (36).

We did not date the mummy using radiocarbon 14 because it was forbidden to take samples from the completely wrapped mummy to preserve its integrity. Based on its burial cemetery era, and mummification style revealed by CT scanning: crisscross wrappings, amulets first used in that era (Two-finger amulet), and cartonnage style, the mummy was suggested dating to the late Ptolemaic era.

## 5. Conclusion

Modern technology using medical CT scanners and 3D printing allows virtual exploration of unknown ancient Egyptian mummy stored at the museum. The methodological approach to virtual unwrapping of the unknown mummy reveals detailed information about its personal profile, dating, health status, social status, rituals and beliefs. The non-invasive technology provides unique opportunity to learn more about life and death in ancient Egypt and opens new perspectives in the study and display of ancient Egyptian mummies.

## Data availability statement

The original contributions presented in this study are included in the article/Supplementary material, further inquiries can be directed to the corresponding author.

## Ethics statement

This study was approved by the Egyptian Ministry of Tourism and Antiquities.

## Author contributions

SSa was responsible for the conception and design, acquisition of data, analysis, interpretation of data, as well as writing the manuscript, generation of the figures, and accountable for accuracy and integrity of the work. Me-H and SSe contributed to interpretation of the results and agreed to be accountable for the integrity of any part of the work. All authors read and approved the manuscript.

## Footnotes

1
http://www.slicer.org
