# Supplementary figures and images for: Scanning and three-dimensional-printing using computed tomography of the “Golden Boy” mummy
Source: Front Med (Lausanne). 2023 Jan 24;9:1028377. doi: 10.3389/fmed.2022.1028377 (PMC9902354; doi:10.3389/fmed.2022.1028377)

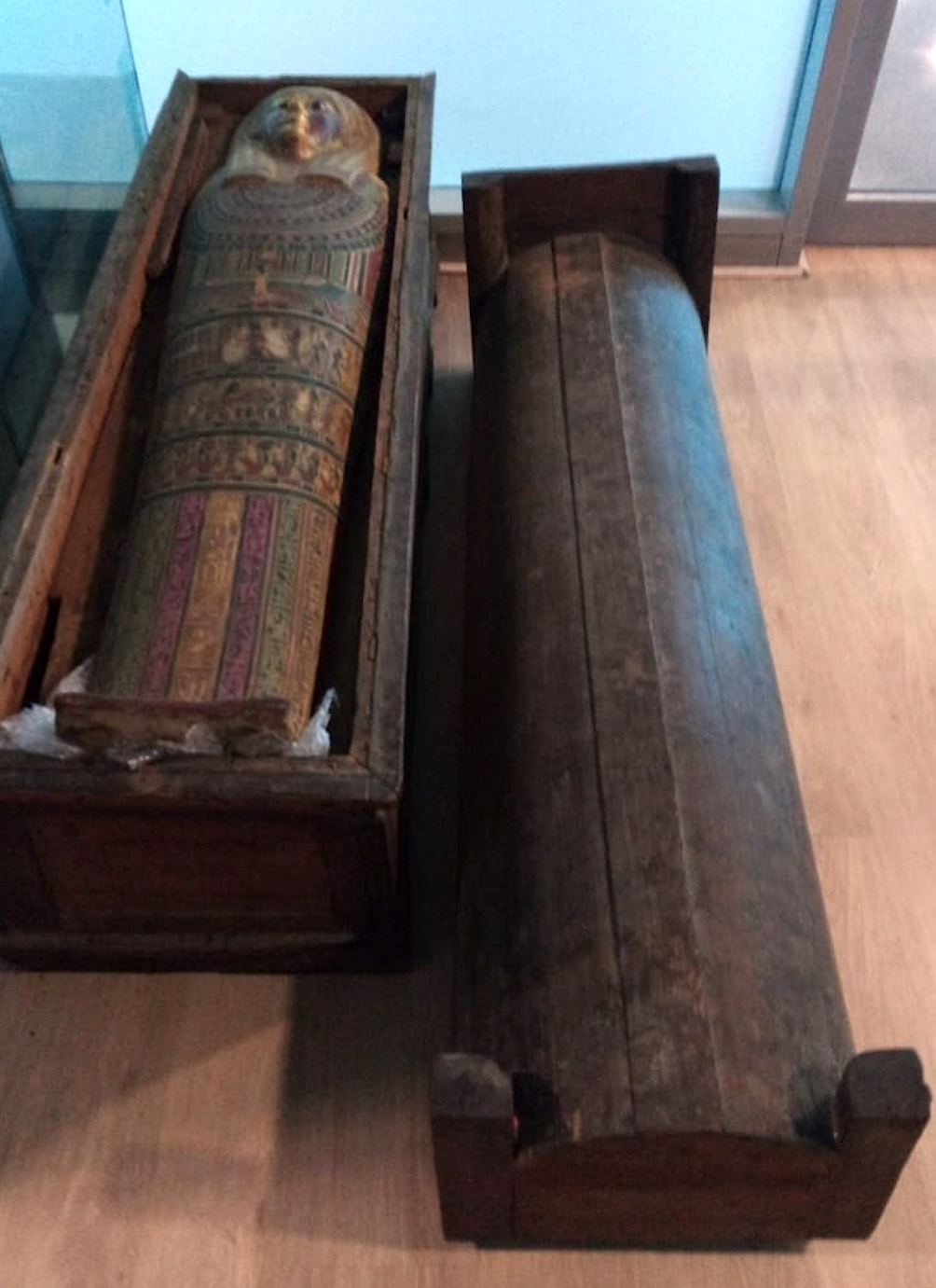

Supplement: Supplementary file 1 [file Image_1.JPEG]

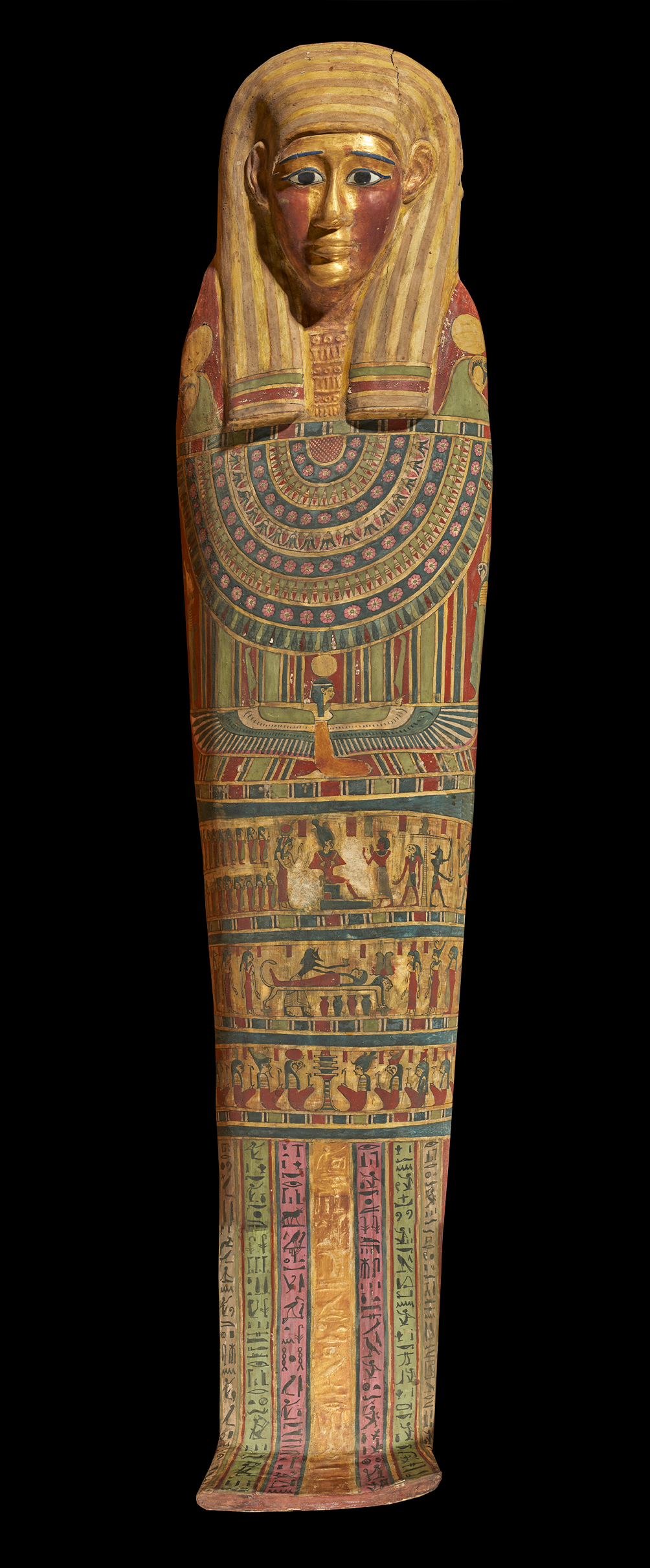

Supplement: Supplementary file 2 [file Image_2.TIF]

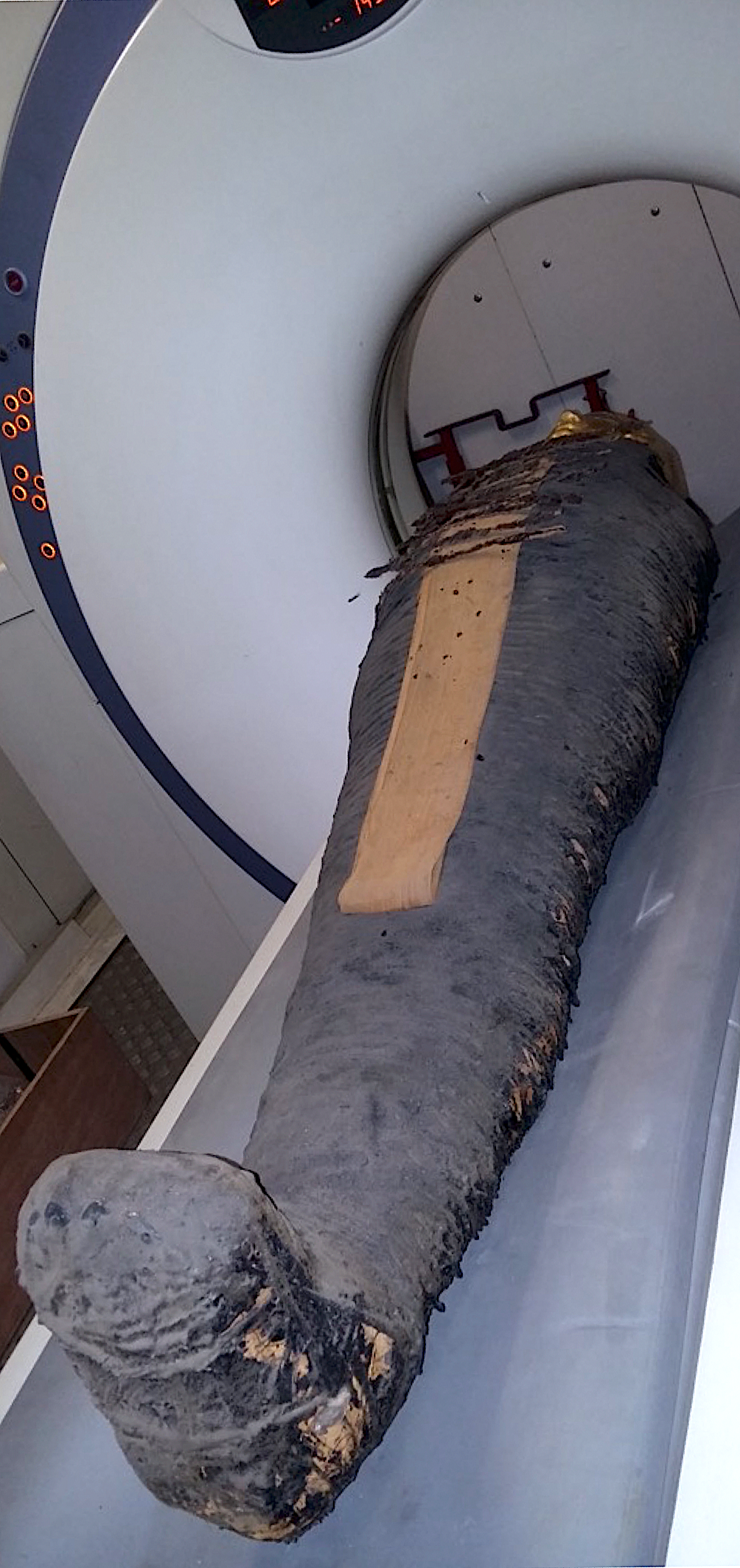

Supplement: Supplementary file 3 [file Image_3.TIF]

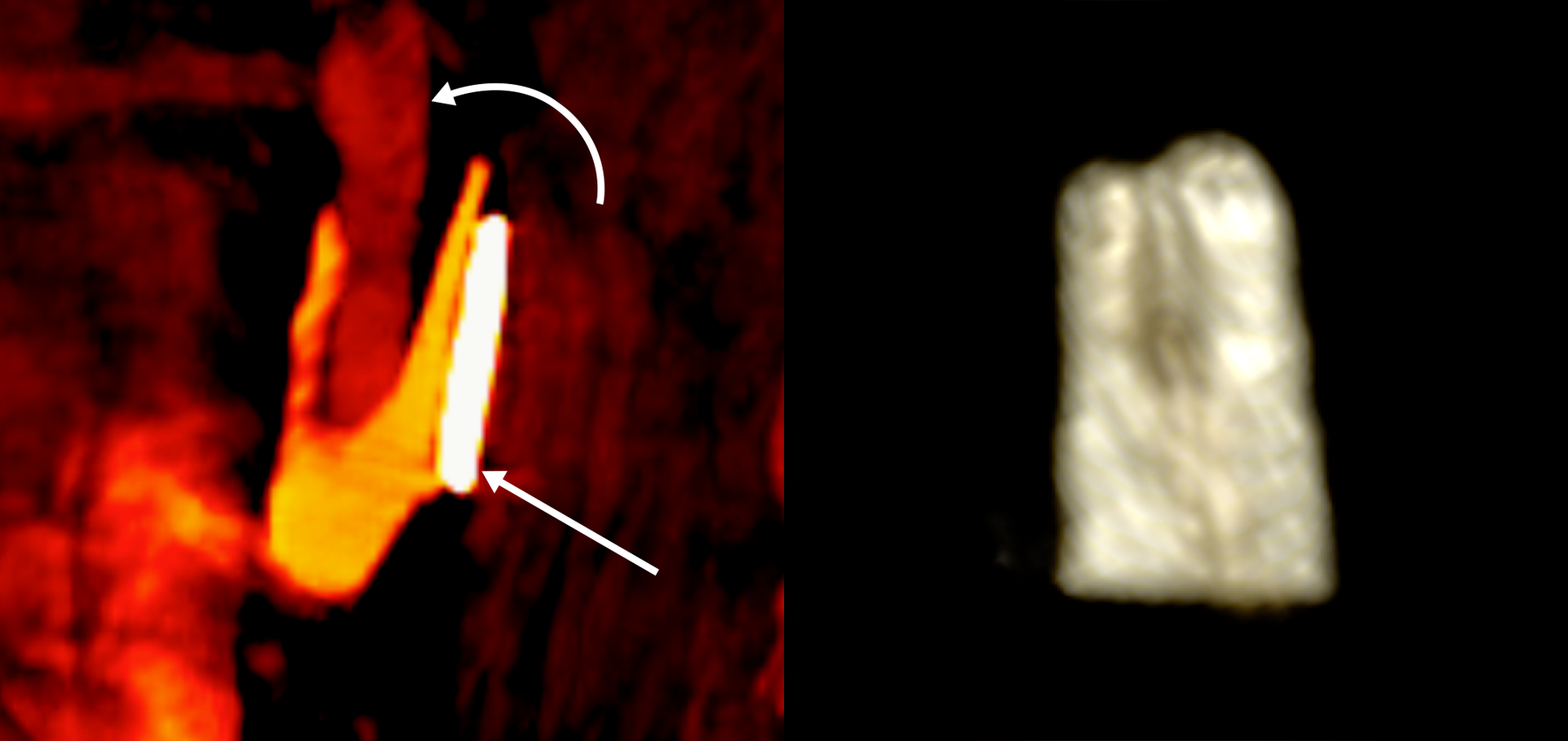

Supplement: Supplementary file 4 [file Image_4.TIF]

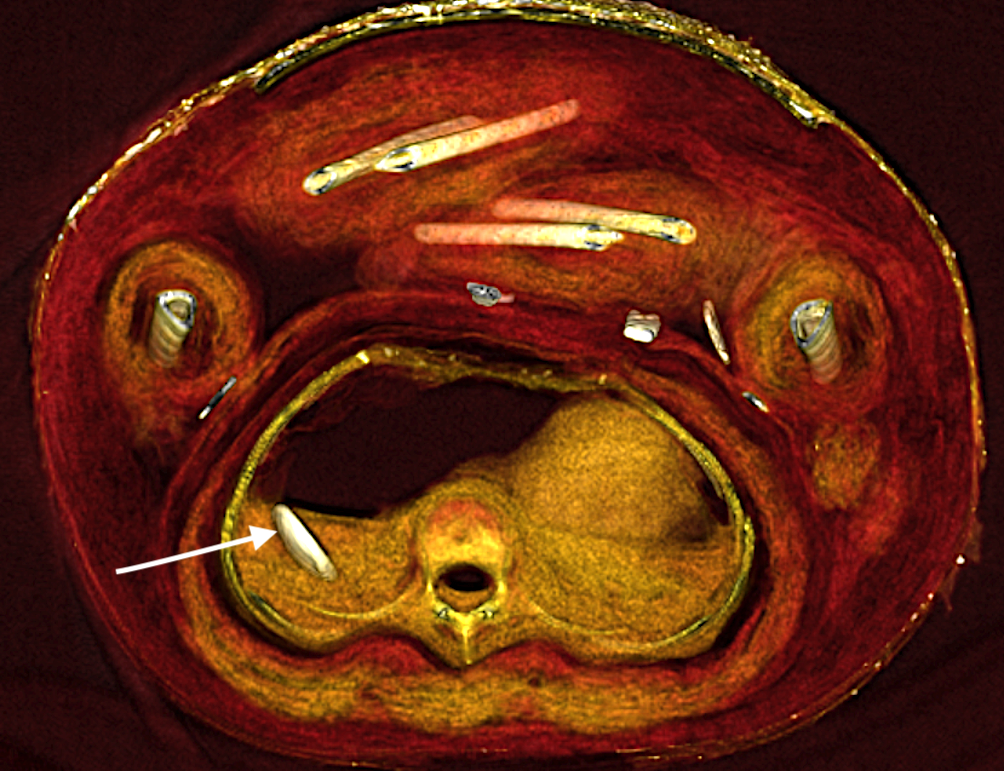

Supplement: Supplementary file 5 [file Image_5.TIFF]

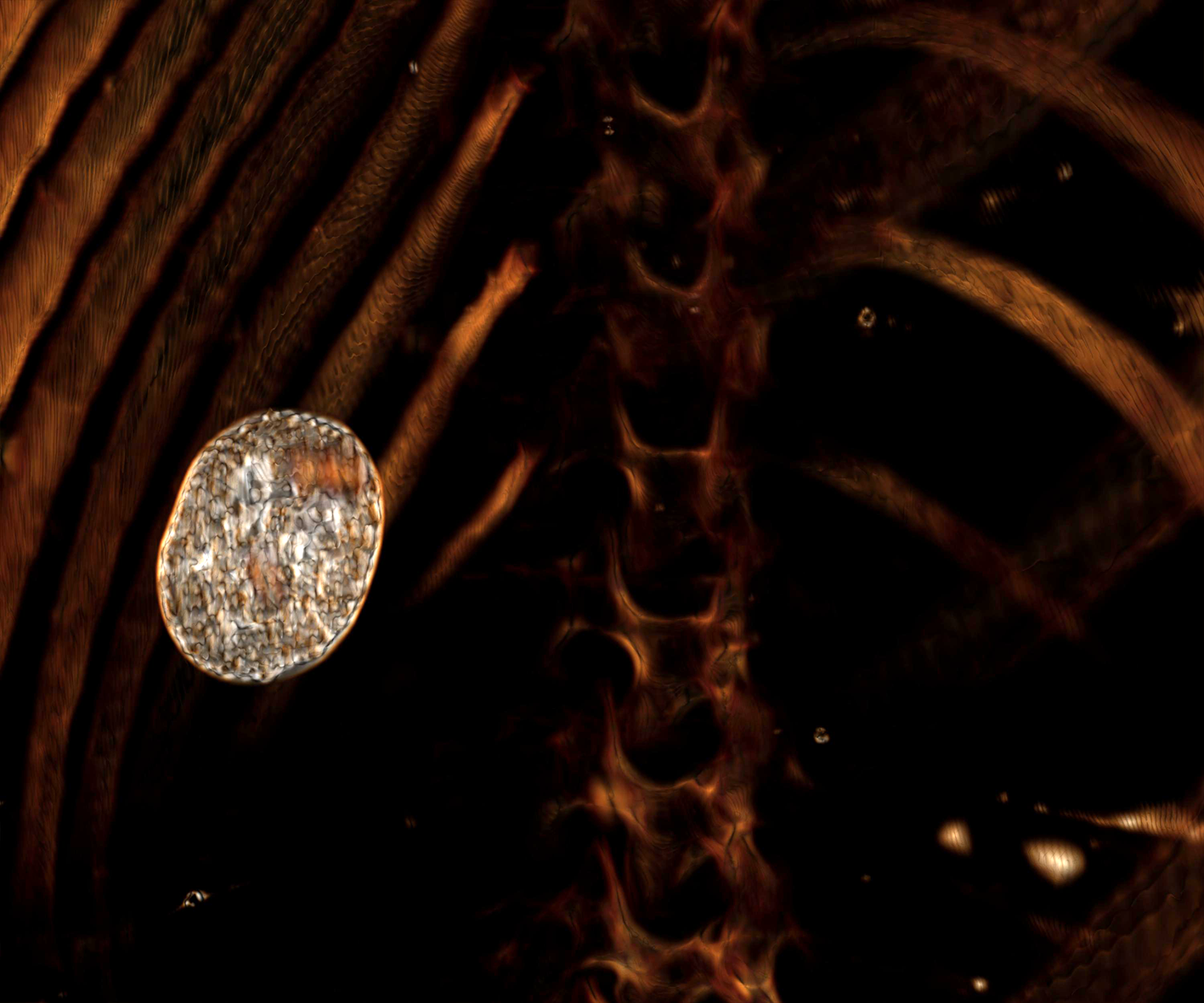

Supplement: Supplementary file 6 [file Image_6.TIF]
