# Supplementary material for: Scanning and three-dimensional-printing using computed tomography of the “Golden Boy” mummy
Source: Front Med (Lausanne). 2023 Jan 24;9:1028377. doi: 10.3389/fmed.2022.1028377 (PMC9902354; doi:10.3389/fmed.2022.1028377)
Supplement: Supplementary file 7 [file Data_Sheet_1.docx]

Legend of Figures and Supplementary Material

# Supplementary Figures

**Legend of Supplementary Figures**

**Supplementary Figure 1:**

A photo of the outer coffin of the mummy stored in Cairo Egyptian Museum in Location SS 17B under the code: CG 31153/TR 21/11/16/16, SR 11413. The non-decorated wooden coffin has a barrel arch shape with a lid that is opened and placed beside the coffin. An inner colored coffin is seen inside the opened outer coffin


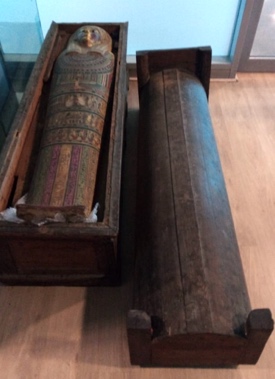


**Supplementary Figure 2**

A photo of the front of the colored inner wooden coffin of the mummy coded TR 21/11/16/16 at the Cairo Egyptian Museum.

The coffin is anthropoid in shape. The head of the coffin has a tripartite wig colored in stripped yellow and beige. The face is gilded; however, the chicks have faded. The eyes and eyebrows are inlaid. The collar consists of nine alternating lines of rosettes, lotuses (opened and closed), geometric lines, and eye-drop shaped.

The chest of the coffin is covered with a drawing of collar with green falcon-headed terminals (smsh) wearing solar crowns.

The abdomen region of the inner coffin shows the drawing of a seated winged Isis wearing a solar crown, followed by drawings of three scenes from ‘The Book of the Dead’ transversely oriented. The top register is the scene of ‘Weight Of The Heart’ from Chapter 125. At the far left, Anubis is seen weighing the deceased heart against Maat feather of justice, while the deceased is watching the process. Following, Horus is introducing the deceased who is adoring to a seated Osiris depicted at the middle of the register. Behind Osiris (to the right of the register) stands Isis wearing crown of solar disc with two horns of a cow. Behind Isis, depicted 16 deities of the other world standing into two lines.

The middle register represents a scene of mummification from (Chapter 72) of ‘The Book of the Dead’. At the center of the register is the deceased lying on a mummification table, below of which four canopic jars. A priest wearing Anubis head mask holds incense burner and performs funeral ceremony. At the right and left side of the mummified deceased stand (seven) deities including Isis, Nephtis, Neith, and others.

The bottom register represents a scene of ‘Fixing The Foot’ in front of Osiris in the lifeafter from Chapter 82 of ‘The Book of the Dead’. At the center stands djed pillar; on both sides several (eight) deities are squatting including Osiris, Horus, Isis, Nephtis, Maat, and Neith

The drawings of the coffin at the legs region show vertically oriented hieroglyphic verses divided into seven columns depicting spells from ‘The Book of the Dead’ (Chapters 82 to 85). The central columns gilded; the lateral columns are colored in pink, green, and grayish respectively from inner to outer.


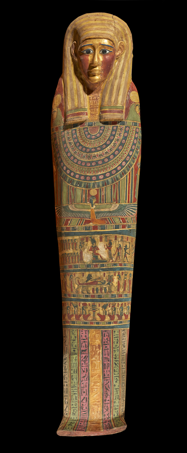


**Supplementary Figure 3**

A photo of the mummy coded TR 21/11/16/16 at the Cairo Egyptian Museum.

placed inside the portable CT scanner in the garden of Cairo Egyptian Museum


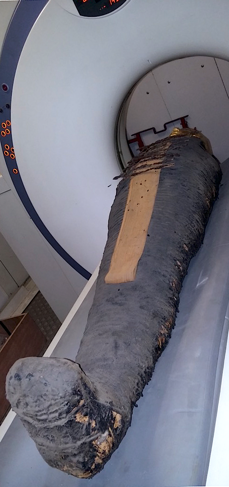


**Supplementary Figure 4**

CT imaging of the Two-finger amulet inside the mummy coded TR 21/11/16/16 at the Cairo Egyptian Museum.

On the left side: A coronal oblique CT image of the pelvic region shows the Two-finger amulet (straight arrow) beside the wrapped penis (curved arrow)

On the right side: A 3D CT image of the Two-finger amulet in the shape of the index and second fingers of the right hand showing detailed lines of the nails


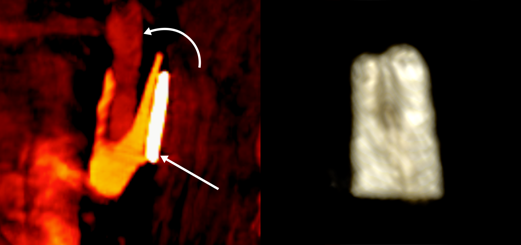


**Supplementary Figure 5**

Axial CT image of the torso of the mummy coded TR 21/11/16/16 at the Cairo Egyptian Museum. The arrow points to a discoid amulet embedded within resin material inside the torso cavity of the mummy

**
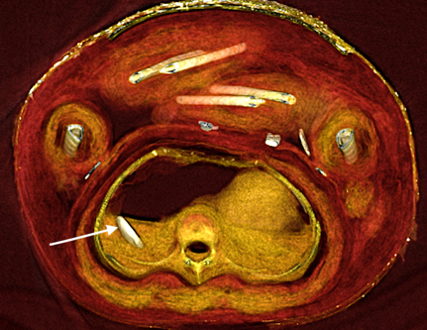
**

# Supplementary Figure 6

Coronal CT image of the torso of the mummy coded TR 21/11/16/16 at the Cairo Egyptian Museum shows a discoid amulet within the chest likely represent a heart scarab


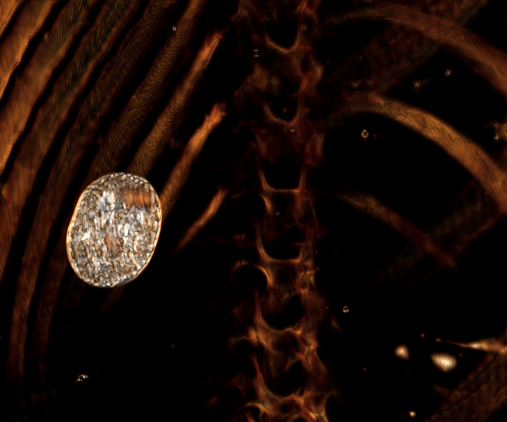


# Supplementary Table

Title: Ancient Egyptian amulets morphology and function

| Name of amulet | Morphology | Function |
| --- | --- | --- |
| Udjat | A human eye with a horizontal cosmetic line that extends above it and a vertical extension below the pupil | The amulet represents the god Horus and represents the healing power. |
| Scarab/Scaraboid | A disc or discoid object symbol of god Kheper and the beetle which propelled the sun across the sky. | Amulet has a remarkable power of protection, transformation and resurrection |
| Flask/Bottle | Flask/bottle amulet is an oblong object | Represents the situla, an ritual metal bucket used to carry holy water in sacred activities. A Nou flask amulet has a rounded bottom |
| Djed | A pillar with transverse processes | Amulet represents the backbone of god Osiris to ensure permanence and revival of the deceased |
| Ajet | A base and a circle in the middle. | Amulet represents the horizon of the sun; the circle in the middle is the sun and the figures at the base the mountains. It symbolizes creation and rebirth |
| Isis-Knot (Tyt) | A rounded solid loop at the top, two arms curved down in the middle, and a base. | The amulet brings the power of Isis in protection of the body. |
| Double Plumes | Double falcon plumes and double ostrich plumes): A flat piece with a central groove delineating two feathers. In double falcon plumes, the feathers are straight; in double falcon plumes the top of the feather is curved outward. | Double plumes signify protection of Amun-Ra god. The two feathers represent the two lives, spiritual and material |
| Two-finger | A flat piece with a central groove delineating two fingers the index and the middle (longer). | The amulet was placed on the lower left of the torso to protect the embalming incision |
| Placenta | A rounded structure with transverse lines | Amulet represents a placenta or sieve the hieroglyph for phonetic letter (kh) |
| Right-angle | Right angle-shaped | Amulet represents a tool used by architect for levelling a building site. The amulet brings balance and levelling to the deceased |
| Tongue leaf | In the shape of a leaf/foil in the mouth. | Amulet represents tongue to ensure that the deceased could speak in the afterlife |
| Lunar crescent | A crescent-shaped amulet | Represents magic, fertility, healing, and motherhood of goddess Isis |

**Udjat amulet:** A human eye with a horizontal cosmetic line that extends above it and a vertical extension below the pupil. The amulet represents the god Horus and represents the healing power.

**Scarab/scaraboid:** A disc or discoid object symbol of god Kheper and the beetle which propelled the sun across the sky. It has a remarkable power of protection, transformation and resurrection

**Flask amulet:** Flask/bottle amulet is an oblong object represents the situla, an ritual metal bucket used to carry holy water in sacred activities. A Nou flask amulet has a rounded bottom

**Djed:** A pillar with transverse processes represents the backbone of god Osiris to ensure permanence and revival of the deceased

**Ajet:** A base and a circle in the middle. Represents the horizon of the sun; the circle in the middle is the sun and the figures at the base the mountains. It symbolizes creation and rebirth

**Isis knot (Tyt):** A rounded solid loop at the top, two arms curved down in the middle, and a base. The amulet brings the power of Isis in protection of the body.

**Double-Plumes** (Double falcon plumes and double ostrich plumes): A flat piece with a central groove delineating two feathers. In double falcon plumes, the feathers are straight; in double falcon plumes the top of the feather is curved outward. Double plumes signify protection of Amun-Ra god. The two feathers represent the two lives, spiritual and material

**Two-Finger**: A flat piece with a central groove delineating two fingers the index and the middle (longer). The amulet was placed to close and bring protection to the embalming incision. (usually placed on the lower left of the torso)

**Placenta**: A rounded structure with transverse lines represent a placenta or sieve the hieroglyph for phonetic letter (kh).

**Right-angle amulet:** Right angle used by architect for levelling a building site. The amulet brings balance and levelling to the deceased

**Tongue leaf:** In the shape of a leaf/foil in the mouth. To ensure that the deceased could speak in the afterlife.
